# In-Line Analysis of Organ-on-Chip Systems with Sensors: Integration, Fabrication, Challenges, and Potential

**DOI:** 10.1021/acsbiomaterials.0c01110

**Published:** 2021-06-16

**Authors:** Stefanie Fuchs, Sofia Johansson, Anders Ø. Tjell, Gabriel Werr, Torsten Mayr, Maria Tenje

**Affiliations:** † Institute for Analytical Chemistry and Food Chemistry, Graz University of Technology, Stremayrgasse 9, A-8010 Graz, Austria; ‡ Department of Materials Science and Engineering, Science for Life Laboratory, Uppsala University, Box 35, 751 03 Uppsala, Sweden

**Keywords:** TEER, ECIS, electrochemical sensors, optical sensors, microphysiological systems

## Abstract

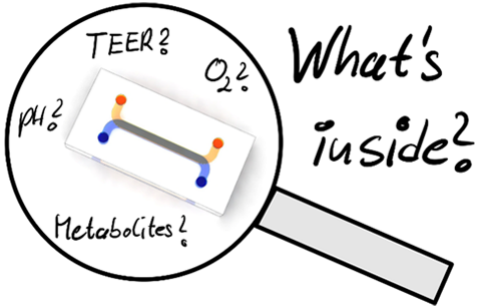

Organ-on-chip systems are promising new *in vitro* research tools in medical, pharmaceutical, and biological research. Their main benefit, compared to standard cell culture platforms, lies in the improved *in vivo* resemblance of the cell culture environment. A critical aspect of these systems is the ability to monitor both the cell culture conditions and biological responses of the cultured cells, such as proliferation and differentiation rates, release of signaling molecules, and metabolic activity. Today, this is mostly done using microscopy techniques and off-chip analytical techniques and assays. Integrating *in situ* analysis methods on-chip enables improved time resolution, continuous measurements, and a faster read-out; hence, more information can be obtained from the developed organ and disease models. Integrated electrical, electrochemical, and optical sensors have been developed and used for chemical analysis in lab-on-a-chip systems for many years, and recently some of these sensing principles have started to find use in organ-on-chip systems as well. This perspective review describes the basic sensing principles, sensor fabrication, and sensor integration in organ-on-chip systems. The review also presents the current state of the art of integrated sensors and discusses future potential. We bring a technological perspective, with the aim of introducing in-line sensing and its promise to advance organ-on-chip systems and the challenges that lie in the integration to researchers without expertise in sensor technology.

## 1 Introduction

Organ-on-chips (OoCs) are microfabricated cell culture platforms capable of recapitulating the function and structure of human organs. This research field has developed over the last 15 years, when the first papers on cells cultured in microfluidic systems were presented in the literature,^1−4^ and it has seen a rapid increase during the last ten years, when the specific term *organs-on-chip* was introduced in the milestone *Science* publication from 2010.^5^ Today, models of human organs such as the heart, lung, liver, brain, and skin have been presented. Often, the purpose of the developed OoC is to improve the quality of *in vitro* testing, for example in the drug development process, by providing *in vivo* like conditions for the cultured cells. Likewise, OoCs can be used as research tools in fundamental medical research in order to understand the mechanisms of disease onset and progression. The reason for the increased interest in OoC is also found in the potential to reduce animal testing, which is problematic due to ethical concerns and the different pharmacokinetic and toxicological effects of drugs on different organisms.

Although 3D cultures of cells and tissues have become mature, analysis of the cell status and the response of the cells to certain stimuli is often limited to optical and fluorescence microscopy using stains and labels. The major drawbacks of these methods are that only a single measurement is possible and often requires the termination of the experiment. Moreover, labels can interact nonspecifically with cells and substances under test. Other analytical techniques such as high performance liquid chromatography (HPLC) are not suitable due to the low sample volumes available. Label-free and continuous real-time analysis of cell viability parameters remains one of the most important unresolved technical challenges in advancing OoC models.

In fact, OoCs are highly suitable for sensor integration as they are normally fabricated using the same micromachining or prototyping techniques that can be used to define and integrate miniaturized sensors.

This review addresses the capabilities of sensor integration for direct access to information about the culture conditions of the cells, the cell proliferation rate and cellular responses to external stimuli, or release of signaling molecules. There have been a large number of review articles published recently on similar topics, focusing either on specific types of sensors,^6^ specific analytes,^7,8^ and general applications of OoCs and disease modeling^9−11^ or giving a brief overview of the many publications available describing OoCs with integrated readout.^12^ What we aim to achieve with this perspectives review is to give a clear introduction to *all aspects of sensor integration*, starting with a fundamental understanding of the sensing principles and fabrication aspects of OoCs. OoC is an interdisciplinary research field, and in addition to holding expertise in a scientific niche, we believe it is important to build general knowledge on all other areas covered to advance the field. To achieve this, we have structured the article to start with an introduction to the different sensing principles that can be integrated in OoCs. The article then includes a section on fabrication aspects that need to be carefully considered when choosing a sensing scheme and ends with a description of solutions presented in the literature. To focus the scope of the review, we have utilized the definition of organ-on-chip systems developed by the EU ORCHID project, stating that “an Organ-on-Chip (OoC) is a fit for purpose fabricated microfluidic-based device, containing living engineered organ substructures in a controlled micro- or nanoenvironment, that recapitulate one or more aspects of the dynamics, functionality and (patho)physiological response of an organ *in vivo*, in real-time monitoring mode”^13^ meaning that we have focused the review on publications where cells are cultured in a microphysiological system, i.e. under flow and in a miniaturized format. On some topics this has however not been possible, and for those cases, articles describing results from macroscale cell cultures have been included, together with a note on this deviation. To further focus the review, we have only considered articles published during the first ten years of history of the OoC research field, i.e. between 2010 and 2020.

Finally, the review article also addresses challenges met when integrating sensors in OoCs which must not be overlooked for successful implementation. It is our ambition that this review article will inspire the development of new OoC models with integrated sensors benefiting the whole scientific community.

**Figure 1:**
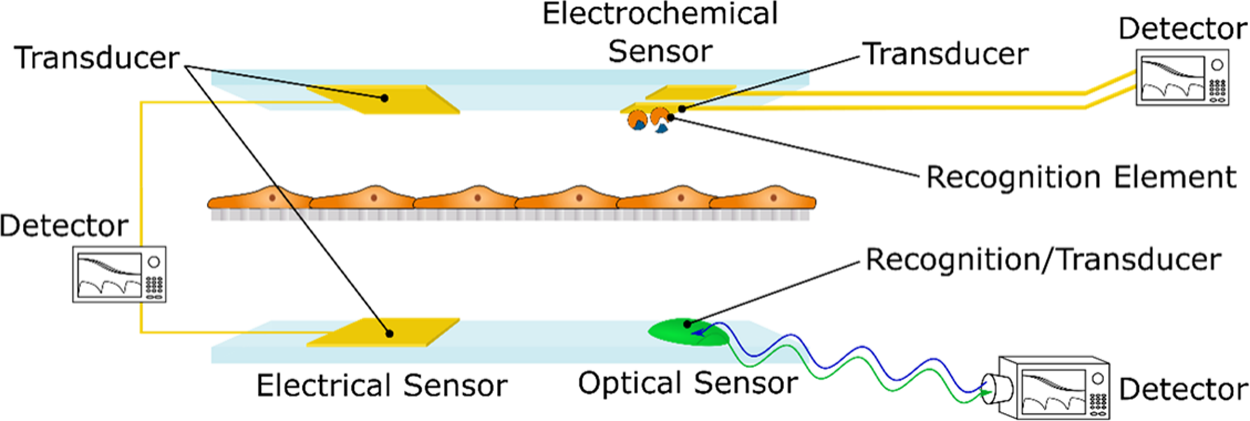
Sensors comprise three parts: a sensing element, or receptor (recognition of the analyte or cell event), a transducer (translating the recognition event into a signal, *e.g*. potential, current, impedance, or optical properties), and a detector (detecting the signal) that is coupled to electronics for signal processing. These parts are depicted here for an electrical sensor, an electrochemical sensor, and an optical sensor, respectively.

## 2 Basic Sensing Principles

In general, sensors comprise three parts; a sensing element, signal transducer, and detector as shown in Figure 1. Sensors integrated into OoCs can be classified into three different groups, depending on their sensing principle; electrical, electrochemical, and optical. Optical and electrochemical sensors are most often used to detect chemical signals (released by the cells or introduced into the OoC as external stimuli or trace elements), whereas electrical signals are most often used to monitor cell growth and mechanical responses. In the following sections, the details of the different sensing principles for each individual sensor category are described.

### 2.1 Electrical Sensors

Electrical sensors are the most commonly used category of sensors in OoCs, possibly due to their simplicity of integration and the comprehensive experience in the field of microelectronics with the integration of miniaturized electrodes. Measured voltages at the electrodes can determine cell properties such as tight junction formation in cell barriers or cell morphology, and physical properties such as strain, which may be used to monitor the contraction of heart cells.

#### Cell Impedance

**Figure 2:**
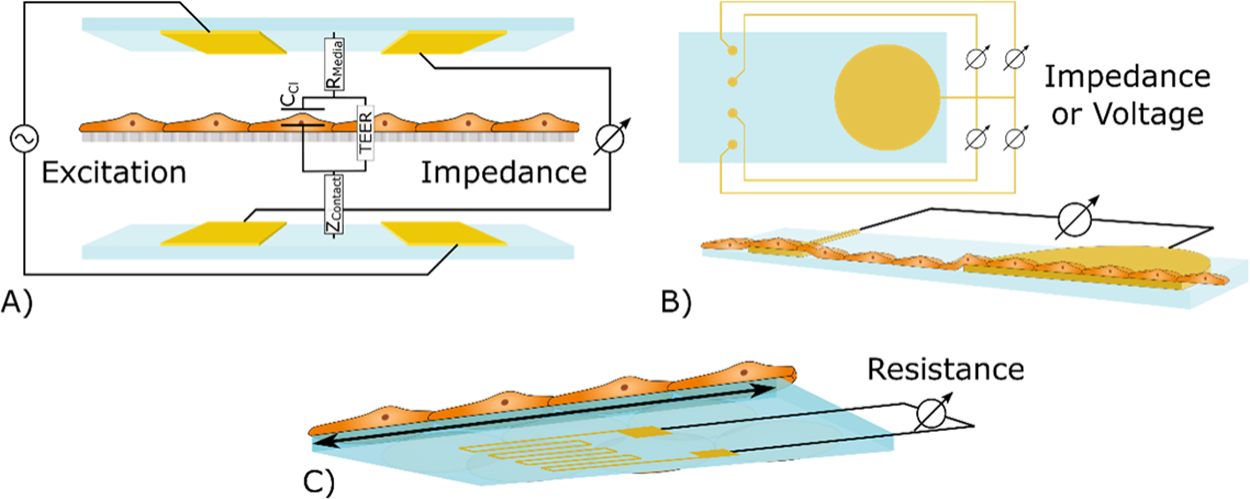
Schematics illustrating the basic function of (A) a TEER sensor where electrodes measure the resistance or impedance of a cell barrier on a membrane in between the electrodes with an overlaid circuit model and (B) an ECIS or field potential sensor showing multiple sensing spots and a larger reference electrode. ECIS characterizes the cells on the small electrodes by impedance measurements, while the field potential sensors measure the extracellular voltage of the cells on the small electrodes. (C) A strain gauge measures the deformation of the substrate imposed onto an electrically conducting element, which changes its resistance with deformation.

The most common electrical sensing in OoCs is trans-epithelial/endothelial electrical resistance (TEER). TEER refers to the resistance obtained between electrodes on either side of a semipermeable membrane on which a biological barrier is formed by culturing endothelial or epithelial cells (Figure 2A). TEER quantifies the integrity of the barrier where a high TEER value is indicative of tight junction formation, which ensures good *in vivo* translation of permeability studies. Through proper modeling, TEER measurements may also be used to monitor other aspects of the biological barrier such as cellular differentiation.^14,15^

TEER is obtained either as the *absolute value* of the impedance measured at a selected single frequency or from an *impedance spectrum* by fitting the collected data to a circuit model and deducing the cell resistance, *R*_cells_. In both cases, the cell resistance is normalized by multiplication with the membrane area and is presented in the units Ω cm^2^.

The most simplistic impedance model of a cell monolayer is a lumped-element model comprising a capacitor, *C*_cells_ in parallel to a resistor, *R*_cells_. This originates from the capacitive behavior of the cell membrane and the resistive paths in-between the cells. In addition to this, it is common to add the resistance of the cell media, *R*_media_, and the impedance related to the contacts between the electrodes and the cell media, *Z*_contact_, to give a good representation of impedance data.

Cell impedance, together with the impedance originating from interaction between the cells and the substrate that they are grown on may also be evaluated by culturing cells directly on a substrate with patterned electrodes. This is often referred to as electrical cell–substrate impedance sensing (ECIS) based on the early work by Giaever and Keese.^16^ In addition to tight junctions, this method is used for evaluating cell attachment, growth, morphology, function, and motility. ECIS utilizes the fact that impedance increases as the electrode size is reduced. An ECIS system therefore includes small working electrodes and a large common counter electrode with negligible impedance (Figure 2B). The measurements are localized, as opposed to TEER, and by using an array of small electrodes, it is possible to find the impedance as a function of location. The absolute impedance is often evaluated at a selected frequency where the change in impedance observed during the experiment is large enough to give a qualitative measure of changes in cell activity.^17^

#### Extracellular Field Potential

In electrically active cells, such as cardiomyocytes and neurons, the depolarization and repolarization of the cell membrane results in changes in the extracellular field potential that may be recorded by voltage sensing electrodes. Multielectrode arrays (MEAs) refer to an array of isolated microelectrodes that can be used for spatiotemporal mapping of the field potentials or, more explicitly, to monitor the occurrence of voltage peaks above a set threshold value. *In vitro* cells may be cultured directly on the MEA surface, and as the potential drops rapidly with distance, a high spatial resolution is achievable. Using this platform, it is possible to measure both the high frequency field potentials displayed by individual cells as well as the low frequency variations representing coupled cell activity and overall organ physiology.^18^ The spatiotemporal data may be analyzed through a number of parameters including field potential duration, peak-to-peak interval, and conduction velocity to characterize the electrophysiological response of the cultured cells.^19^

#### Strain

A strain gauge measures mechanical deformation, *e.g*. bending of a cell culture membrane, which could be correlated to certain cell responses such as contraction of cardiac tissue.^20,21^ The sensor typically consists of a passivated conductive meander attached to a surface. A change in resistance is measured as the meander is elongated or compressed along the conductive paths (Figure 2C). As the change in resistance is small in comparison to the actual resistance, the sensor is often connected to a Wheatstone bridge, which converts it into a difference measurement and thus improves the accuracy.^22^

### 2.2 Electrochemical Sensors

Electrochemical devices transform the effect of an electrochemical interaction between an analyte and an electrode into a read-out signal.^23^ The read-out signal is either a current flowing between electrodes or a potential difference between electrodes, and the most common analytes are oxygen or pH. An important utilization of electrochemical sensors are biosensors, in which the recognition element makes use of a biochemical mechanism.^24^ Both potentiometric sensors and amperometric sensors can be used as biosensors.

#### Potentiometric Sensors

Potentiometric sensors work by measuring potential differences between a reference electrode and an indicator electrode. There are a few devices commonly used as potentiometric sensors; metal oxide based (MOx) sensors and ion-sensitive field-effect transistors (ISFETs).

MOx sensors commonly measure a difference in potential between a working electrode and a reference electrode. The potential of each electrode can be expressed by the Nernst equation, and the potential differences are dependent on, for instance, the pH of the solution. The reference electrode is commonly silver coated with silver chloride (Ag/AgCl), and the working electrode is a metal oxide from, e.g., manganese, zinc, ruthenium, tungsten, or iridium.^25^ The equilibrium state at the metal oxide surface is affected by the pH of the solution, leading to changes in surface potential and electrical properties of the working electrode, which has a known dependence on the pH of the solution.^26^

**Figure 3:**
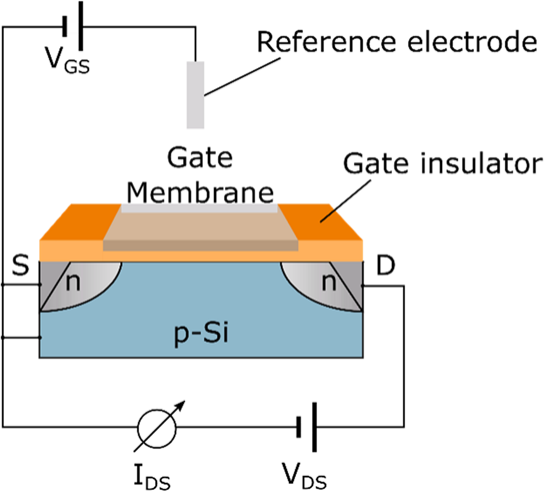
Schematic drawing of an ISFET used for electrochemical measurements. A voltage (V_DS_) is applied between the source (S) and the drain (D). The resulting current (*I*_DS_) is the signal. An ion-sensitive membrane or receptors for biomolecules are added to the top of the gate. The concentration of the ions or charged biomolecules influences the electric field across the gate area and hence the current *I*_DS_.

An ISFET is a potentiometric device that employs an ion-sensitive membrane on a field-effective transistor (FET) and a reference electrode. ISFETs are generally fabricated on a silicon substrate.^25^ In the setup, a voltage is applied between the source and the drain, creating a channel under the gate area, where a current can flow (Figure 3). This electrical current is controlled by the electric field generated at the gate and influenced by charged species above the gate. Thus, by adding an ion-sensitive membrane or molecular receptors to the top of the gate area, the concentration of the respective ions and charged biomolecules can be determined by measuring the current passing through the transistor.^10,26,27^ The latter approach is an example of a biosensor.

#### Amperometric Sensors

In amperometric sensors, a potential is applied between a working electrode and a reference electrode. Electrochemically active species in a solution are then detected through changes in the current flowing between the electrodes. Frequently, a third auxiliary electrode (sometimes called the counter electrode) is used to increase the long-term stability of the reference electrode by reducing the amount of current passing through the reference electrode and protecting it from changing half-cell potential. In most amperometric setups, the reference electrode is made of Ag/AgCl, and the counter electrode is a conducting material, usually Au or Pt. The working electrode must be made of a chemically stable and conductive material, such as Pt, Au, or carbon-based materials^28,29^ that support electrochemical reduction and/or oxidation activities on its surface. As the analyte undergoes an electrochemical reaction on the working electrode, a current will form, giving the output signal. If the potential between the working electrode and reference electrode is maintained at a constant value, the technique is termed *amperometry*. If the voltage is scanned between two predetermined values, the measurement technique is called *voltammetry*.^28,29^

By applying a biological recognition element to the working electrode, amperometric sensors can function as indirect sensors for nonelectrochemically active analytes.^28^ A well-known example is enzyme-based glucose sensors, where an immobilized enzyme (glucose oxidase) catalyzes the transformation of glucose into hydrogen peroxide and other products. By measuring the current generated from the formed hydrogen peroxide, the glucose concentration can be determined.^30,31^

### 2.3 Optical Sensors

Optical sensors are based on detecting changes in an optical property, such as luminescence, absorption, refractive index, or scattering. Refractive index and scattering have so far not been explored for OoC. A major benefit for all optical sensors is that the read-out does not require physical contact between the sensing element and the detector. Instead, the signal can be transferred directly in the transparent microfluidic chip or through a window.^32−34^

#### Photoluminescence

**Figure 4:**
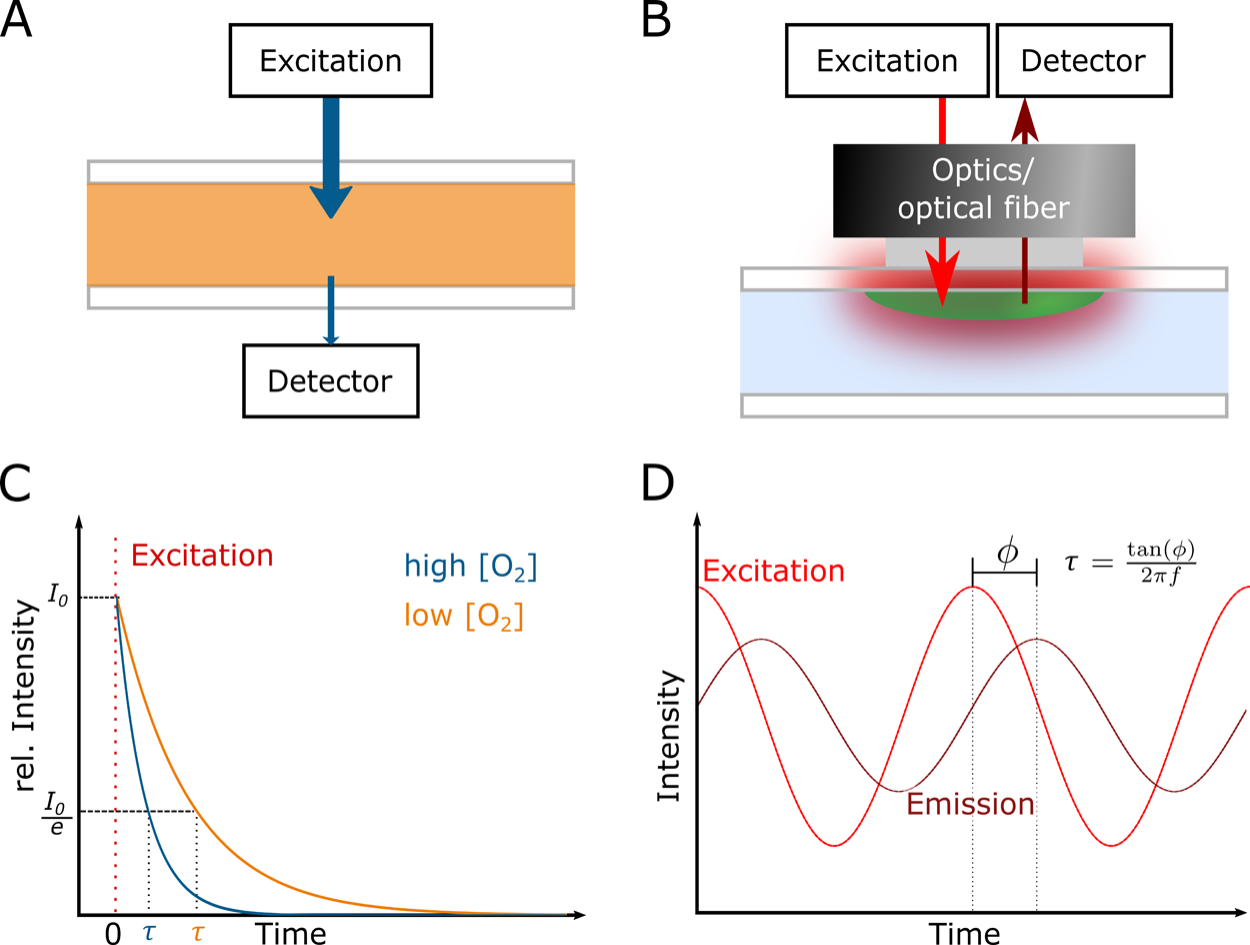
Schematic drawing of the optical sensor setup based on absorption (A) and luminescence (B). Absorption measurements are usually performed in transmission mode. Nonabsorbed light that reaches the detector on the other side is detected (A). The excitation light source and the detector are usually mounted on the same side in luminescence based measurements (B). The used signal is either the intensity or the luminescence lifetime which both can be affected by interactions between the dye and the analyte (C). The luminescence lifetime is often determined via phase modulation (D). This method uses an amplitude modulated light source to excite the indicator, and the detected signal shows a phase shift relative to the excitation. The phase shift ϕ is related via  to the lifetime τ, where *f* is the frequency of the modulated signal.^30^

Photoluminescence is a term encompassing fluorescence, phosphorescence, and delayed fluorescence. Typically, a luminescent sensor consists of a sensing element inside the microfluidic device, as well as a light source and a detector, which are both mounted externally (Figure 4). In most cases, additional filters are added to separate background luminescence, excitation, and emitted light. Lenses and waveguides can be included in the setup to increase efficiency of excitation and collection of emitted light. The sensing element consists of a luminescent indicator dye that is sensitive to the target analyte and a polymer matrix that hosts this dye. The choice of indicator dye and matrix defines the sensor properties and is therefore crucial for the sensor specifications, including targeted analyte, measurement range, sensitivity, and optical setup. The sensing element can also include other components, such as enzymes and inert reference dyes.^35^ Sensor components including dyes, matrixes, and additional components are extensively reviewed elsewhere.^35−38^

When luminescent molecules are excited by light, they emit a photon. This process is characterized by luminescent intensity and lifetime, which are the two different measurement types of luminescent sensors. Here, lifetime is defined as the average amount of time a fluorophore remains in the excited state before emitting a photon. Other molecules that are in close vicinity to the sensing element can interact with the luminescent indicator dye resulting in amplification or quenching of the luminescence. In microfluidics, oxygen sensors are the most successfully applied luminescence sensors. These are based on dynamic quenching, which affects both luminescent intensity and lifetime.^37^ Luminescent intensity can be measured using a standard fluorescence microscope which enables a spatially resolved measurement. However, intensity measurements are prone to errors caused by ambient light, inhomogeneities in the illumination field and indicator distribution, as well as bleaching of the luminescent molecules. Ratiometric methods, like dual-wavelength rationing, or dual lifetime referencing, using an inert reference dye, can be used to improve intensity-based measurements.^39,40^ Lifetime measurements can be accessed by pulsed excitation using single photon counting or phase modulation^41^ (Figure 4) and are less prone to errors because the lifetime is an intrinsic property of the indicator molecule.

Luminescent indicator dyes are available for some specific analytes, such as oxygen, pH, and ions. Alternatively, an indirect sensing method can be applied, similar to electrochemical sensing. A biological recognition element is incorporated that catalyzes the conversation of the target analyte, and a side product is detected by the indicator dye.^36^ Furthermore, luminescent sensors can also be used for temperature measurements as both the luminescent lifetime and intensity are influenced by temperature.^35^

#### Absorption Measurements

Absorption measurements use indicator dyes that change their absorption spectrum upon interaction with an analyte. They can be performed using a simple setup including a light emitting diode (LED) to illuminate a dissolved indicator dye in the microfluidic channel. The signal, in terms of light intensity, is detected on the opposite side of the channel at a specific wavelength using an optical filter (Figure 4).^42,43^ The detected absorption depends on the optical path length along the microfluidic channel, the concentration of analyte, and the molar absorption of the dye, according to the Beer–Lambert Law. Realization of an absorption sensor in miniaturized OoC systems is complicated by the small size of the systems. However, the method can be used for absorption of light by Phenol Red to determine the pH of cell culture media.^42,43^

## 3 Integration of Sensors–Miniaturization

**Figure 5:**
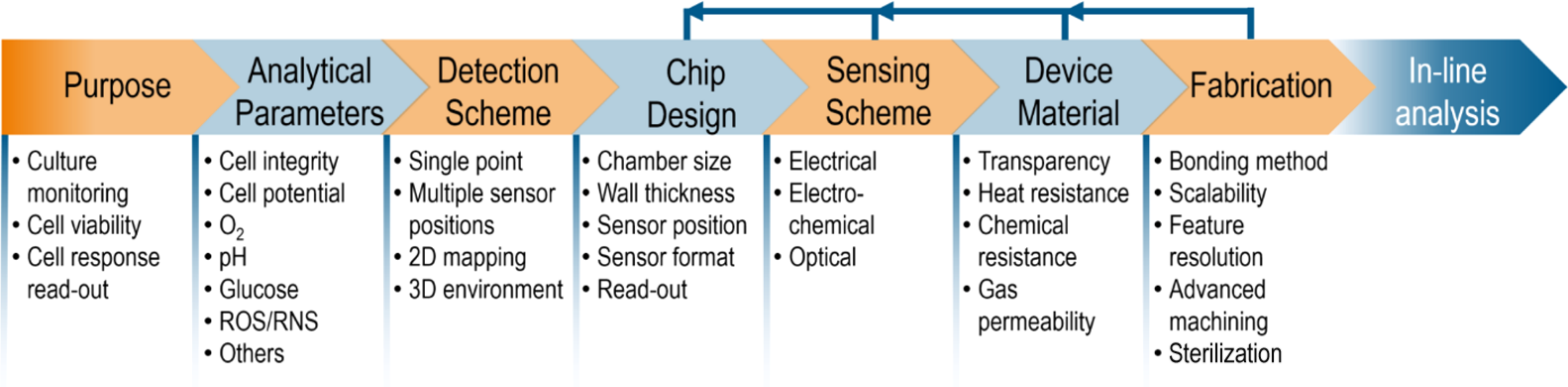
Flow diagram describing the path toward robust sensor integration in organ-on-chip devices. First, the purpose of the experiment has to be defined, and relevant analytical parameters identified. Accordingly, the detection scheme has to be chosen. To obtain this, the chip design, sensing scheme, device material, and fabrication, which all are dependent on the initial decisions, have to be adjusted in an iterative process, because some points exclude others. The main aspects of the flow diagram are elaborated with specific points below, which serve as a guideline.

Electrical, electrochemical, and optical sensors all face different challenges related to their integration in OoC systems. The following section discusses the practical aspects of fabrication and integration, material selection, design, and known pitfalls. Figure 5 shows a flowchart of important aspects to consider when designing OoCs with in-line sensors.

### 3.1 Integration of Electrical Sensors

**Figure 6:**
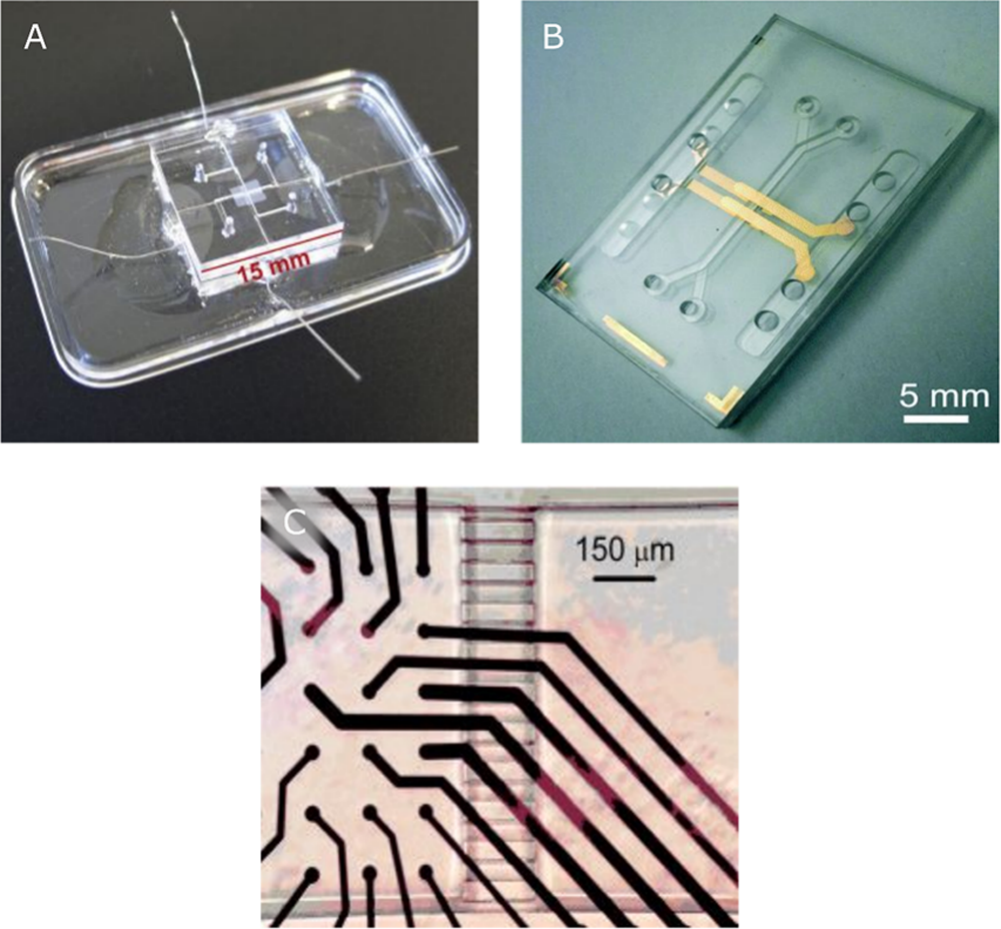
Examples of integrated electrodes: (A) wire TEER electrodes (Reprinted and adapted with permission from ref (58). Copyright 2016 Elsevier), (B) thin-film TEER electrodes (Reprinted and adapted with permission from ref (61). Copyright 2019 The Royal Society of Chemistry), (C) MEA electrodes (Reprinted and adapted with permission from ref (62). Copyright 2018 Elsevier).

Microelectrode integration has been studied and optimized for more than 50 years within the field of micro-electro-mechanical systems (MEMS) and microelectronics. Examples of integrated electrodes can be seen in Figure 6. When electrodes are integrated into OoCs to serve as sensors, attention needs to be given to the choice of electrode material and the design of the electrodes.

#### Electrode Material

A number of different electrode materials have been proposed for integrated electrical sensors in OoC systems. The choice of material depends on the requirements of operation, *e.g*. low impedance of the electrode–electrolyte interface, operating frequency, mode (AC or DC), and biocompatibility.

The most frequently used metals, Au and Pt, are polarizable. This means that charging effects occur at the interface between the solution and the electrode, resulting in a large contact impedance, especially at low frequencies. Cell impedance measurements are negatively affected when the background becomes much larger than the signal to be detected. The exact details of the charging effects are not yet fully understood, but many physical models are proposed to describe the effects of the charge build-up.^44^ How the charging effects are dealt with is important to consider when designing a cell impedance sensor. Fortunately, many successful routes to overcome this issue have been presented. One approach is to use impedance spectroscopy, which sweeps from low (Hz) to high frequencies (MHz) and uses a circuit model to subtract the impedance originating from the electrode–electrolyte interface.^45^

Increased electrode area is another approach to circumvent the issue of high contact impedance. This can be achieved in different ways, either by simply increasing the electrode footprint or by maintaining the small footprint and fabricating the electrode such that the surface area is enlarged, *i*.*e*. using a material with high porosity or surface roughness. One commonly used material is Pt-black, which can be applied as an extra layer on top of a thin-film electrode fabricated in Au^19^ or Pt.^46^ Porous materials that compromise the whole electrode can be porous Au^47^ and the conducting polymer poly(3,4-ethylenedioxythiophene):polystyrenesulfonate (PEDOT:PSS).^48^

Ag/AgCl electrodes are nonpolarizable, which gives a lower contact impedance between the electrode and the solution compared to polarizable materials. Therefore, Ag/AgCl electrodes are suitable also for measurements using DC and AC at low frequencies. Ag/AgCl wire electrodes are commercially available in standardized formats due to their frequent usage within the field of electrochemistry. This simplifies their access and integration.

It is imperative to make sure that the used materials are not harmful to the cells. This is especially important to consider for electrode materials in direct contact with the investigated cells. Metals such as Cu where the ions have harmful effects on cultured cells are therefore not a suitable choice.^49^ Care must also be taken when using the popular electrode material Ag/AgCl as silver ions have known cytotoxic effects.^50^ Other electrode materials that are more inert and are considered biocompatible, include Pt, Au, ITO, Ti, and TiN.

#### Electrode Fabrication

Control over electrode size and position can be easily obtained if thin-film electrodes are integrated. Here, materials such as Pt or Au are often used.^51^ Fabrication of thin-film electrodes requires the conducting material to be deposited onto the substrate using techniques such as evaporation or sputtering. The metals can either be deposited directly on the substrate^19^ or using an additional adhesion layer such as Ti.^46,52^ Moreover, the electrodes are commonly patterned to a desired design and shape by photolithography, using either a lift-off technique or etching. Photolithography gives micrometer resolution, allows for wafer-scale fabrication and enables alignment of multiple thin-film layers on the same device.^53^ Thin-film electrodes are normally attached to hard substrates such as glass or Si wafers, but there are examples where the electrode material rests on soft and stretchable substrates.^54,55^ In such cases, it is particularly important to assess the long-term stability of the electrodes as they can be damaged by movement. Compared to other techniques for fabricating electrodes in OoCs, which are discussed below, thin-film technology is superior in achieving precise and repeatable electrode patterns and is therefore advantageous for generating desired electric fields and providing a high signal-to-noise ratio (S/N).

Simplified fabrication techniques that do not require cleanroom processing could in many cases be more practical, *e.g*. screen printing or direct laser printing.^56,57^ Evaporated electrodes could also be patterned using a shadow mask during deposition.^15^ An alternative to self-fabrication may be to order custom patterns from printed circuit board (PCB) manufacturers. The standard is to use Cu as a conductor in electronics; however, these may be covered with an Au layer to improve the biocompatibility of the chips.^47^

In many early works, electrodes were obtained by inserting Pt^58^ or Ag/AgCl wires.^59,60^ These are still used, as thin films may be too fragile on soft materials such as polydimethylsiloxane (PDMS). In addition, wires are commercially available in a range of set dimensions and the technical requirements for the fabrication are much lower. The main disadvantages, however, are the reduced precision and repeatability, difficulty in scaling up fabrication and the reduced S/N for large electrode separations. In the most common configuration, wires are inserted on the sides of the microfluidic channels, which results in a large cell-to-electrode distance. Douville et al. instead positioned two Ag/AgCl wires directly over and under the cell layer to measure TEER impedance spectra, which showed a more uniform electric field across the cell layer.^60^ In the latter case, the S/N was significantly higher than in systems where the wires were further away.

An electrical passivation layer is sometimes needed to limit the active electrode area, *e.g*. in MEAs, or completely hinder electrical currents between the cell media and the electrode, *e.g*. in strain gauge resistors. The passivation layer could in principle be any biocompatible dielectric material of sufficient thickness that can be attached to the surface, often with the added requirement that it must be possible to micropattern the layer. Examples of materials used for passivation layers are SiO_2_, Si_3_N_4_, PDMS, polyimide, and parylene.

#### Electrode Design Considerations

Cell impedance is measured using either two or four electrodes. In a four-point measurement, one electrode pair applies a current and the other pair measures the resulting voltage drop. A four-point measurement greatly reduces the influence of the contact impedance compared to a two-point measurement, as a measurable voltage drop is only available along the common path of the two electrode pairs, *i.e.*, across the sample.

For TEER measurements, which aim to measure the impedance of all cells on a membrane simultaneously, the electric field across the cell membrane should be as uniform as possible. The easiest way to achieve this is to place identical electrodes above and below the cells, ensuring that the electrodes cover an area equal to the membrane. Uniform electric fields can also be achieved by concentric or interdigitated electrodes.^63^ If electrodes are put in the microfluidic channels with a large cell-to-electrode distance, the obtained TEER could be overestimated, which is important to keep in mind when interpreting data from the literature comparing the tightness of different cellular barriers. It should be noted that the TEER is mainly overestimated at low TEER values. However, this could be corrected by theoretical calculations of the error to find the geometrical correction factor for the given geometry.^14^

Optical access could be an issue when electrodes are placed above or below cells as transmission microscopy is often essential for cell characterization. To circumvent this, transparent electrode materials, such as indium tin oxide (ITO)^64^ may be used. Another approach is to observe cells next to or in-between the microstructured electrodes.

### 3.2 Integration of Electrochemical Sensors

#### Electrode Materials and Integration Methods

In many ways, integration of electrochemical sensors is similar to that of the electrical sensors discussed above. The same materials and fabrication techniques are often applied. Typically, the working electrodes and counter electrodes are made from Au or Pt, and reference electrodes from Ag/AgCl. The reference electrode can be integrated by a galvanic process.^65−67^ MOx can be deposited by sputtering of *e.g*. RuO_2_/RuO_4_,^68^ and ZnO,^69^ or electrodeposition methods, *e.g*. IrO_2._^66,67^ The ISFET, which is a semiconductor device, is commonly fabricated on Si wafers by standardized processes used for complementary metal oxide semiconductor field effect transistors (CMOS).^70^ A semiconducting channel is formed between two electrodes (source and drain). Contrary to CMOS, the gate, which regulates the conductivity of the semiconducting channel, is covered with an ion-sensitive membrane. The ion-sensitive membrane in ISFETS is commonly made of either Si_3_N_4_ or Ta_2_O_5_ which can be deposited by thermal growth, sputtering, or chemical vapor deposition (CVD).^71−75^

#### Electrochemical Biosensors

Electrochemical biosensors utilize a biological recognition element, such as enzymes, proteins, antibodies, or receptors, which needs to be immobilized on the electrode surface, and are mostly based on amperometric sensors. Enzymes are commonly immobilized in polymer hydrogels. This involves covalently bonding the enzyme to the polymer, for instance with glutaraldehyde, or physically entrapping the enzyme in the polymer. The hydrogel/enzyme matrix can then be either physically adsorbed to the electrode surface, or, in most cases, cross-linked on the electrode. These procedures have been used to immobilize enzymes in poly(2-hydroxyethyl methacrylate) (pHEMA),^67,76^ Nafion,^66,77^ and cross-linked bovine serum albumin (BSA)^31^ to measure glucose and lactate, and additionally glutamine and glutamate.^76^ A different approach has been presented by Giménez-Gómez et al., who electrodeposited pyrrole and glucose oxidase on an electrode.^65^ Electropolymerization is a useful technique for immobilization of enzymes and straightforward to apply. It has also been reported that glucose oxidase, lactate oxidase, choline oxidase, and l-glutamate oxidase can be immobilized on the inside surface of an SU-8 microreactor with the surfactant Triton-X for measurements downstream of a cell culture plate.^78^ Another method to integrate electochemical biosensors is presented by Bavli et al., who embedded commercial sensors for glucose and lactate in a PMMA flow-chamber.^30^

Antibodies or aptamers can be immobilized on the working electrode by covalent bonding. Reported procedures employ a self-assembled monolayer (SAM) on the electrode and subsequently apply carbodiimide coupling (NHS/EDC) to immobilize the recognition element, i.e. the antibody/aptamer. This method has been used in combination with electrochemical impedance spectroscopy to bind commercially available antibodies or aptamers for detection of albumin, α-glutathione-S-transferase, and creatine kinase.^43,79,80^ Another method using amperometric detection has been reported by Riahi et al.,^81^ who utilized magnetic microbeads to perform on-chip immunoassays. By binding primary antibodies against transferrin, and albumin biomarkers to the microbeads, they could reduce the limit of detection (LOD) compared to off-chip systems. This is due to the increased surface area allowing for a greater number of recognition elements to be bound.^81^ Another advantage of immobilizing antibodies to microbeads is the increased flexibility regarding the analyte. By exchanging antibodies on the microbeads, the same system can be used to detect other molecules.

### 3.3 Integration of Optical Sensors

To perform measurements with luminescent sensors inside OoCs, a sensing element needs to be integrated inside the device. The matrix of the sensing element, together with the position and format of the sensing element, determines which integration method can be used.

#### Sensor Format

**Figure 7:**
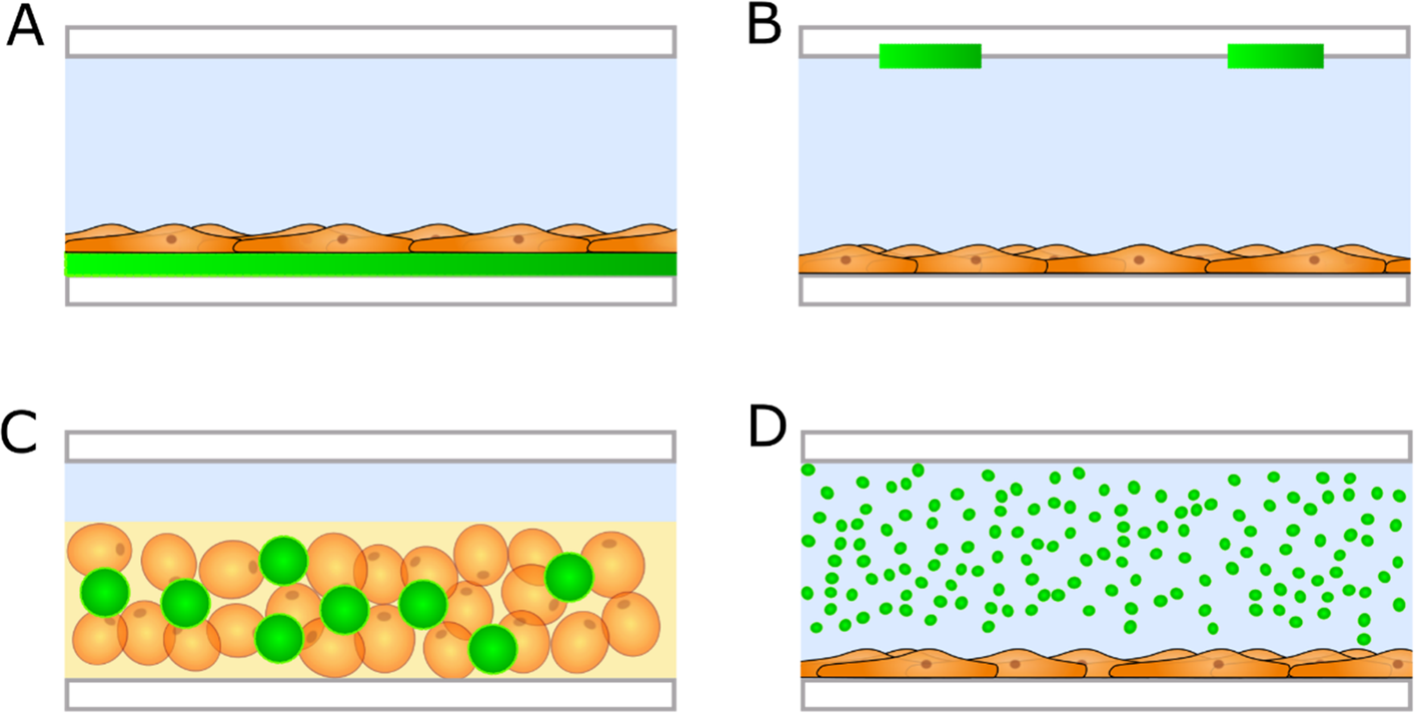
Schematic drawings of different luminescent sensor formats (green layers and dots) used in an OoC. Films (A) and spots (B) can be placed on the outer walls of the chip and below the cells. Sensor particles can be integrated within hydrogel cell constructs (C) or dispersed in the cell culture medium (D). It is also possible to dissolve hydrophilic indicator dyes directly in the cell culture medium without additional components.

The sensing element can be integrated as a thin-film, patterned film (spot), or bead (Figure 7). Each of these formats has specific advantages and disadvantages. Spots are used for single-point measurements, while thin-films allow for 2D mapping of gradients along the cultured tissue.^40^ Sensor spots can be restricted to the area where the measurements are needed and are therefore less prone to interfere with the cells or other measurement methods. Structures down to 5 μm in size have been reported but require highly sophisticated methods for preparation.^82,83^ Furthermore, highly sensitive read-out instruments are necessary when the sensor element is spot-patterned, as the signal strength decreases with decreasing spot size. Both sensor films and spots are fixed on a surface in the cell culture system. Sensor beads allow more flexible ways of integration and placement, which can be beneficial in more complex systems. Sensor beads are immobilized in a hydrogel for the integration inside the OoC, and sensor beads can even be incorporated in the same hydrogel in which the cells are cultured.^30^ This approach allows for 3D measurements and mapping of gradients directly inside the cell culture constructs, although the presence of the sensor beads inside the hydrogel might disturb the cells.

Other approaches use sensor dyes that are dissolved in the culture medium or sensor beads that are dispersed in the culture medium for measurements (Figure 7). These approaches do not require techniques to integrate the sensors inside the microfluidic device and are therefore easy to apply. However, the sensing element is not fixed in these approaches, which might cause problems with signal intensity and stability, and possibly interfere with the cultured cells.

#### Sensor Integration

There are several methods in which optical sensing elements have been integrated into microfluidic systems and to date not all of these sensors have been used in OoCs, although the methods are potentially useful for OoC systems as well.

**Figure 8:**
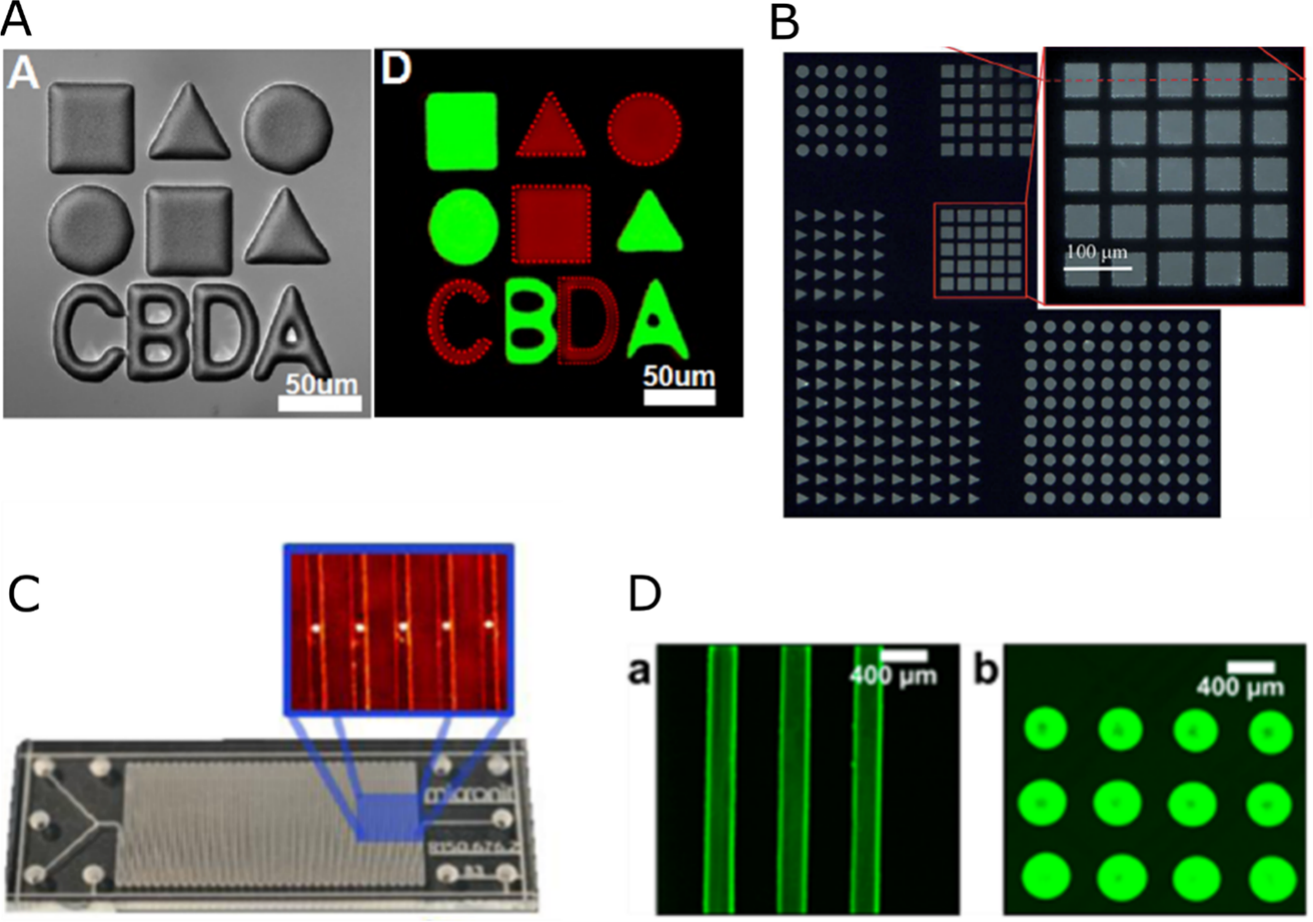
Examples of sensor spots patterned with (A) reactive ion etching (RIE) (Adapted with permission from ref (87). Copyright 2012 Elsevier), (B) laser ablation (Adapted from with permission from ref (88). Copyright 2014 The Royal Society of Chemistry), and (D) microdispensing (Adapted with permission from ref (91). Copyright 2014 American Chemical Society). (C) Spots can also be formed inside a closed microfluidic device using photopolymerization (Adapted with permission from ref (94). Copyright 2016 Springer).

Spin- and knife coating are well-known approaches for creating controlled thin matrix films with materials commonly used in OoC fabrication, *e.g*. polystyrene (PS) or PDMS, but also with other polymers.^82,84−87^ The films can be patterned to create sensor spots of any shape using dry etching after deposition of a protective mask or maskless laser ablation (Figure 8A, B).^82,87,88^

Likewise, sensor formulations can be coated on defined substrate areas using a shadow mask. In addition, features of 100 μm have been achieved using masks in combination with spray coating.^89^ Good adhesion of the sensor matrix to the substrate is necessary to prevent detachment of the sensing element, which is especially important when fabricating very small features. Wet etching^90^ or powder blasting^89^ are used to roughen the surface of the substrate before the sensor formulation is applied to promote adhesion.

Direct writing techniques do not require masks and can be used to form sensors directly inside unassembled, open microfluidic channels. Ehgartner et al. used an airbrush mounted on a computerized numerical control (CNC) machine to create sensor spots with a diameter of 2 mm.^89^ More commonly used are piezoelectrically actuated microdispensers which are capable of depositing single drops of a liquid^91−93^ (Figure 8D). Pfeiffer et al. performed photopolymerization inside a closed commercially available microfluidic chip to form oxygen and pH sensor spots.^94^ Formation of the sensor spot inside the chip, eliminates the need for subsequent assembly steps that could potentially be harmful to the sensor performance (Figure 8C).

Photolithography can be used to pattern the sensing element, if photocurable polymers are used as sensor matrix. This method requires dyes with a high photostability to avoid bleaching. Sensor elements down to a diameter of 5 μm have been fabricated using commercial photoresists.^83,95^ Furthermore, PDMS molds can be used to shape photocurable polymers during the curing step, allowing for the formation of patterns with hydrogels containing different sensor dyes in a single fabrication step.^96,97^

Staining beads or particles, or coupling the indicator dye to the surface is another common practice. Beads can be dispersed in the working fluid for use in microfluidics.^98^ Alternatively, the sensor beads can be immobilized on a surface or incorporated into hydrogels.^86,99−101^ It is also possible to use hydrogels with dye-doped beads to form 3D cell culture constructs or even cell spheroids with integrated sensor beads.^30,34,102^

### 3.4 General Considerations

In the previous parts of this section, integration aspects of the specific sensor types were discussed. Below follow some more general considerations that apply to all the different sensors when they are integrated in OoCs.

#### Sensor Position

The position of the sensor is a very important aspect to be considered when designing the microfluidic device. In principle, sensors can be placed anywhere in the chip, either inside the cell culture area or along the inlet and outlet channel regions. In general, positioning the sensors closer to the cells leads to a more localized measurement compared to placing the sensor further away, *e.g*. in the microfluidic channel. This is especially important if the cells are not homogeneously distributed in the culture area. Large differences in the oxygen distribution along a culture of clustered cells has been shown by spatially resolved measurement.^40^ If spatially resolved detection of the analyte is not possible, careful consideration should be given to both the position of the sensor and the section of the cells that is contributing to the measurement.

Some sensors have special requirements for their placement. Detection of dissolved oxygen or short-lived species, such as reactive oxygen/nitrogen species are measured favorably in close proximity to the cells. Oxygen measurements are prone to inaccuracy due to the oxygen ingress or release from the bulk material used to fabricate the OoC device, which has been reported for bioreactors made from PDMS.^43^ Materials with low gas permeability can be used to avoid oxygenation through the device.^43,100^ Oxygen may also leak into the system via the fluidic tubing and connections, as well as any other external ports, so it is beneficial to place the oxygen sensors close to the cells. Positioning the sensor directly underneath the cells leads to more local detection compared to placing the sensor on top of the medium channel. Other analytes, such as glucose, lactate, and pH are not altered after exiting the cell culture area and are not prone to interference with the OoC device itself. Therefore, the sensing element can be integrated downstream, or in another sensing chip on a microfluidic circuit board. However, the disadvantage to this is that it introduces a delay in the read-out of the measurement, as the analytes need to travel from the cell culture chamber to the sensor.

Electrical sensors of cell properties have special requirements for their placement. The electrodes for TEER measurements should be carefully placed on either side of the cell barrier in order to create an even current density across it. MEAs need to have the cells cultured directly onto the working electrode as close physical contact strongly increases the sensitivity of the measurements. The localized nature of the MEA electrodes enables studies of variations across the cell culture, including the synchronization of cardiomyocytes beating and cell migration. This also means that, in contrast to the integral TEER measurements, MEA sensing is resilient to inhomogeneities such as holes in a cell monolayer through its spatial resolution. For electrochemical sensors, the placement of the working electrode is the most important to consider as it needs to be close to the cells for some analytes, such as oxygen. The counter electrode and the reference electrode can be placed elsewhere on the chip, *e.g*. at the outlet^103^ or downstream from the working electrode.^78^

#### Long-Term Stability

The long-term stability of the sensor is another important aspect to be considered as many OoC systems are used continuously for several weeks. The stability can be influenced by the degradation of the sensing element or by interaction with the exposed environment. Often this variation of the sensor performance over time is expressed as “sensor drift”. In the case of electrical and electrochemical sensors, thin film electrodes and porous materials are sensitive to delamination and fragmentation, which can be induced via physical wear at the contact points between the electrodes and the external read-out or during sensor cleaning. However, the main risk for degradation of electrodes is probably biofouling, *i*.*e*., the adsorption of nonspecific proteins from the cell culture media.^5^ This aspect is relevant for both long-term experiments and devices that are to be used for multiple experiments in which the reference point or background signal can shift in-between measurements. Biofouling does not seem to be reported yet for OoC systems, but considering how extensively it has been studied in other microfluidic systems it bears relevance for OoCs as well. Factors, such as electrode porosity,^104^ cell seeding density, and cell proliferation rate, are known to affect biofouling of integrated electrodes.^65^ To avoid biofouling, antifouling coatings can be applied.^105,106^ Alternatively, measures can be taken to avoid direct contact between cells and electrodes, or electrode cleaning routines can be introduced.^6^

Biofouling also occurs in optical sensors from the interaction with cell media. The effects can be reduced by using a suitable sensor matrix or coatings and placing the sensing element away from the cells.^107^ The photodegradation of the sensor dyes can be an additional limiting factor for long-term stability. Sensor dyes with low photostability degrade upon interaction with light and lose their brightness. This so-called bleaching of the sensor dye is mainly a problem in intensity-based measurement but also affects lifetime-based measurements as the signal strength decreases over time. It can be solved by using dyes with a high photostability and limiting the light exposure of the sensing elements.^37^

Another critical point for long-term stability of optical sensors is the immobilization of the sensor molecules in the sensor matrix. Physical entrapment can cause leaching into the cell culture medium and a deterioration in the sensor performance.^108^ This is especially a drawback with using hydrophilic sensor dyes or enzymes in electrochemical or optical biosensors. Therefore, covalently bonding the molecules to the matrix using larger molecules or even particles that are physically trapped inside the matrix can be favorable to avoid leaching.^36,37^

Another aspect to consider for sensors that utilize enzymes as a biological recognition element is the stability of the enzyme itself. Enzymes degrade over time when they are not stored in the dark or frozen. The conditions in OoC systems are mainly optimized with respect to the cultured cells, *e.g*. by maintaining the temperature at 37 °C and using buffers to control pH. Fortunately, these conditions match well to the optimal working conditions of most enzymes. Consequently, they show a high activity which decreases over time as the enzymes degrade.

#### Chip Assembly

Most methods used to assemble microfluidic chips can influence sensing elements that are already fabricated on one of the chip layers. There are well established methods to seal microfluidic devices with integrated electrodes, and structured thin-film electrodes are rarely affected by standard bonding techniques such as thermal bonding or adhesion bonding. Electrochemical biosensors and optical sensors on the other hand are more heavily influenced by the bonding process. Forming the sensing element of optical sensors inside the closed device as the final step in the fabrication is always favorable but very rarely reported.^94,109,110^

All in all, it is difficult to give general recommendations on which bonding method is suitable for optical sensors as the stability of the sensing element depends on the respective sensor dye and matrix. Methods that exclude the use of heat or solvents are to be preferred and if UV curable glues are used, then prolonged application of UV light should be avoided. Plasma is known to change the matrix surface and thereby affect the sensor properties. However, plasma bonding has been successfully used to bond microfluidic chips with integrated luminescent sensors.^111^ Thermal bonding methods are not suitable for biochemical sensor concepts that include enzymes, either using optical or electrochemical read-outs, because the used enzymes will be degraded by elevated temperatures. The temperature stability of pure optical sensors is defined by the respective sensor dye and matrix. Ehgartner et al. reported on an oxygen sensor that could withstand temperatures (180 °C) that occurred during anodic bonding of a glass-silicon chip,^89^ and there are commercially available sensor foils that state a temperature stability of 120 °C.^112^

#### Complexity and Standardization

An aspect that is important to consider before deciding to integrate sensors in an OoC is the increased manufacturing cost associated with each chip. Although time-resolved and detailed information can be gained using integrated read-out, it may not always be economically justifiable and standard off-chip assays might be sufficiently good in combination with low-cost single-use microfluidic devices. For example, integration of electrodes for high-resolution read-out requires access to advanced microfabrication tools and specialized clean room laboratories. Integration of optical sensors does not require the same level of complex fabrication but specialized know-how in material chemistry and optics is required. The additional cost aspect might not be detrimental for research focused applications but they are crucial for the commercial potential of OoCs with integrated sensors.

Another issue to consider is how much the complexity level is increased by integrating sensor elements into an OoC system, *e.g*. when additional wires, cables, and electronic components have to be implemented. This is in particular the case when multiple OoCs are combined to form specific tissue models, so-called multi-OoC models.

Another point that is important when developing new sensor schemes is the possibility for production scale-up. The sensor integration step must be suitable for mass production of the OoCs. Herein, common chip materials, such as PDMS and glass, are expected to be replaced by thermoplastics due to a better processability among other things.^113^ Sensor integration must be compatible with these materials. Standardization is in general a current challenge in the OoCs field where the microphysiological systems used differ greatly in terms of size, material choices, and design. Furthermore, connection interfaces and software modules have to be compatible with a potential combination of different peripheral instruments. Another issue that is becoming relevant, when OoCs are adapted more and more by industry is sterilization. In research laboratories, sterile conditions are usually obtained by using ethanol or similar agents. Under good laboratory practice (GLP) conditions in industry, sterilization is an important point, and autoclaving, gamma, or beta sterilization are the accepted procedures. These techniques can be harmful to some of the integrated sensors and bias the performance or render them inoperable. This has to be considered already in the development and integration of the sensor for OoCs in the future. These, and other important aspects, related to bringing OoCs to industrial use are thoroughly addressed in a recent review article by Ramadan and Zourob to which the interested reader is referred.^114^

## 4 Examples and Applications of Sensors in Organ-on-Chip Systems

This section addresses reports from the literature whereby electrical, electrochemical, and optical sensors have been integrated into OoC systems or microfluidic-based cell cultures. Note that this is not a complete list, the aim is rather to give a general overview of integrated sensors for OoC applications. While there are reports published on the use of integrated electrochemical and optical sensors, there are substantially more on the use of integrated electrical sensors, building upon the development from the MEMS and IC research fields. Tables 1 and 2 give an overview of existing OoCs with integrated sensors.

**Table 1:** List of Various Applications of Electrical Sensors Used in Organ-on-Chip Models^a^

type and use case	material	organ model + ref
Trans-Epithelial/Endothelial Electrical Resistance (TEER)		
**wire electrodes**	• Ag/**AgCl**	kidney (HREC, MDCK)^59^
cell layer impedance		cell monolayers (bEND.3, MDCK-2, C2C12)^60^
		gut (Caco-2)^14,115^
		skin (HaCaT, U937)^116^
		lung (16HBE14o, hAEpCs, AT II)^117^
**thin-film electrodes**	**• Pt**	BBB (hCMEC/D3)^45,58^
cell layer impedance	**• stainless steel tubing**	BBB (hBMVEC, primary pericytes and astrocytes, hiPSC neurons)^118^
	**• Au**	heart (HUVEC, hiPSC- CM)^19^
	• Ti/Pt/Ti/**Pt-Black**	retina (ARPE-19, HREC, SH-SY5Y)^46^
	• Cr/Au/Ag/**AgCl**	BBB (b.End3, C8D1)A^51^
		blood vessel (bEnd.3)^119^
	Cu/Ni/**Au**	lung (16HBE14o, A549)^47^
	**• stainless steel sheet**	eye (HCK, HCE-T)^120,121^
	• Ti/Au/**Ti**	gut (Caco-2)^15^
	• Ti/**Au**	airway (hAECs) and gut (Caco2)^52^
		lung (HpMVECs HUVECs, A549)^122^
Electrical Cell–Substrate Impedance Sensing (ECIS)		
**thin-film electrodes**	**• ITO-Pt pair**	breast cancer (MDA-MB-231)^64^
		ovary CHO-K1^17^
local cell impedance	**• Au**	placenta (BeWo)^123^
cell adhesion		lung cancer (A549, H1299, H460)^124^^,^^b^
cell contraction		heart (NRVM)^125^
		vessel (BAEC)^43^^,^^b^
		vessel (HUVEC, VSMC, RBL-2H3)^126^
	**• ITO**	lung cancer (NCI-H1437)^127^
	• Ti/**Au**	lung (A549, MRC-5)^128^^,^^b^
		liver cancer (Huh7)^129^
		heart (iPSC-CM)^130^^,^^b^
		breast cancer (MCF-7)^131^
	TiW/**Pt**	heart (HCT-116 eGFP)^132^
**3D electrodes**	**• Au** sheet	liver (HpeG2, HeLa)^133^^,^^b^
cell growth		
cell viability		
Field Potential		
**microelectrode arrays (MEAs)**	• Cr/Au/**PEDOT:PSS**	pancreas (mice islet, human islet)^18^^,^^b^
action potential	• Ti/Pt/Ti/**Pt-Black**	retina (ARPE-19, HREC, SH-SY5Y)^46^
cell contraction	**• PEDOT:PSS**	heart (mESC)^48^^,^^b^
beat rate		brain (hESC Regea 08/023, hiPSC 10212.EURCCs, hiPSC IMR90-4)^134^
	• Pt/**Pt-black**	pancreas (C57BL/6)^62^
		heart (HUVEC, hiPSC-CM)^19^
	**• TiN**	hPSC-CM^135^^,^^b^
		heart (hiPSC-CMs-Cor.4U)^136^^,^^b^
	• Ti/**Au**	brain (rat cortical neurons)^137^
		heart (iPSC-CM)^130^^,^^b^
**3D electrodes**	**• Pt-**PDMS	heart (dissociated from E18 rat embryo heart)^138^^,^^b^
beat rate		
stimulation		
field potential		
Flow Sensors		
**thin film**	• Ti/**Pt**	blood vessel (bEnd.3)^119^
velocity distribution		
Strain Gauge		
**printed**	**• CB:TPU**	heart (NRVM, hiPS-CM)^20^^,^^b^
strain	• Ti**/Au**	heart (NRVM, hiPS-CMs)^21^^,^^b^

a The sensing material or surface is marked bold amongst the adhesion layers or matrix material.

b Well-based model outside of the OoC definition.

**Table 2:** List of Optical and Electrochemical Sensors Used in Organ-on-Chip Models

analyte	method	organ/tissue model (cell type)	note
oxygen	luminescence	gastrointestinal microbiome chip (Caco-2, primary CD4^32^)	in combination with TEER
		liver-on-chip (HepaRG, HUVECs, PBMCs;^33^ HepG2/C3A, HeLa;^34^ HepG2^139^)	
		multiple cell types (A549 human lung carcinoma epithelial-like cell line, primary human ASC, primary human HUVECs^100^)	
		multiple cell types (HeLa, NHDF^40^)	ratiometric measurement, mapping of 2D oxygen gradient
	amperometric	liver-on-chip (human primary hepatocytes^140^)	
			
glucose	amperometric	spherical 3D microtissue (human colon carcinoma cell line HCT116 eGFP^31^)	hanging droplet cell cultures
lactate			
			
oxygen	luminescence (oxygen)	liver-on-chip (HepG2/C3A^30^)	*in-situ* measurement of oxygen
glucose	amperometric (glucose and lactate)		in-line measurement with amperometric sensor
lactate			
			
oxygen	luminescence (oxygen)	liver/heart-on-chip (human primary hepatocytes, iPSC-CMs^43^)	at-line measurement
pH	absorption (pH)		
albumin	immunobiosensor (Albumin, GST-\alpha, CK-MB)		
GST-\alpha			
CK-MB			

### 4.1 TEER Measurements

The only requirement for integration of TEER measurements in OoC is that the electrodes must be in contact with the cell culture medium on both sides of a porous membrane. This means that the integration of TEER measurement is rather straightforward and has been extensively implemented to date. In early work, Ferrell et al.^59^ included Ag/AgCl wires through access holes in the PDMS wall of a microfluidic chip, which modeled the epithelial barrier of the kidney through culturing of either HREC or MDCK cells on the porous membrane suspended inside the chip. Here, microfluidic perfusion was used to induce shear stress levels at *in vivo* conditions (∼1 dyn/cm^2^). The cell barrier integrity and the development of tight junctions (TJs) was confirmed via TEER read-out and immunofluorescence staining for ZO-1 tight junction protein. Further, a “calcium switch” to cell media with low calcium concentration was applied, which is known to disrupt TJs, and TEER measurements were used to follow the loss of barrier integrity minute-by-minute.

Similar approaches for electrode integration have been taken by Ramadan et al. measuring TEER in a skin-on-chip model,^116^ Beißner et al.,^120^ and Mattern et al.^121^ on the DynaMiTES OoC platform where stainless steel electrodes were used instead of Ag/AgCl, expanding the fabrication and design possibilities.

To develop a deeper understanding of TEER measurement techniques in OoC, Odijk et al.^14^ designed a chip to investigate the electrical current distribution over the cell culture membrane area, and its dependence on the width, length and height of the culture chamber as well as the measured TEER value. In their setup, Ag/AgCl electrode wires were inserted in the inlet and outlet channels. They discovered that the current distribution was uneven across the membrane area with higher current densities in the regions closer to the inlet and outlet hosting the electrodes. This results in an overestimation of their on-chip TEER measurements compared to conventional Transwell measurements, especially for long and shallow channels and low TEER levels. They conclude that to compare different systems, a geometrical correction is needed. Further, it is highlighted that TJs cannot be quantified by TEER measurements in nonconfluent cell barriers. More specifically, their model predicts an 80% reduction in TEER caused by only 0.4% uncovered cell growth area. This could in part explain variations in reported TEER values found in the literature. The same group reported an improved TEER setup with four Pt wires, one in each inlet/outlet.^58^ To find the TEER value, they measured across all wire pairs and applied a simple circuit model to deduce the TEER value with Gaussian elimination. Also here, geometrical correction was applied. The advantage of this method is that it allows for compensation of temperature changes, fluid conduction changes, air bubble disturbances, and changes in electrode position, and it significantly improved the TEER measurement quality.

Metal wire electrodes have been popular, possibly due to their ease of integration and adaption to existing OoCs. Douville et al. focused on obtaining a more accurate placement of the Ag/AgCl electrode wires, which are usually manually inserted, above and below the cell culture membrane by casting channels in the PDMS chip specifically for the wires.^60^ Accurate placement increases the S/N ratio by reducing the resistive contribution from the cell media and by providing a more uniform current density across the cell barrier. TEER values were measured for bEnd.3, MDCK-2, and C2C12 cells, with stable results over 7 days. TJ disruption was induced through treatment with Triton-X and resulted in a significant drop in the TEER value. In this work, the TEER values were determined by fitting a circuit model to the measured impedance spectra, as discussed in section 2.1. The impedance spectra were measured between 10 Hz and 1 MHz. Baseline impedance measurements before cell seeding were used to subtract contributions from the cell media and membrane. Although the system generated reproducible and important biological results, it is a disadvantage that the electrodes need to be placed directly on top of the cells, thereby obstructing the optical path required for imaging the cells inside the chip.

Moving the electrode fabrication to thin-film techniques allows for more precise electrode definition, smaller electrode designs and a thinner substrate. The smaller footprint of the electrodes allows for more cell area to be visible, e.g. in the case of very thin and transparent Au electrodes, to observe the cells through the electrode^52^ Yeste et al.^46^ presented a system whereby the TEER electrodes were placed on the bottom of the chip and ARPE-19, SH-SY5Y, and HREC cells, modeling the blood–retinal barrier, were cultured on a “membranelike” grid patterned on the electrodes. This is a very uncommon electrode configuration for TEER measurements, and it yields a read-out at double the actual TEER value as the electric field penetrates the cell layer twice for the measurement. In their circuit model they must also consider leakage currents through the substrate and bottom channel cell media. To validate their method a control experiment was performed, in which a third measurement electrode was added above the cell layer to allow them to compare their data to trans-barrier measurements. Although both the fabrication and measurements in this setup are challenging, it has the advantage that it allows for simultaneous real-time high spatiotemporal resolution imaging and TEER measurements, as the electrodes were integrated in the bottom of the device.

In recent work, van der Helm et al. have provided further advancements in the analysis of TEER values using thin-film electrodes.^15^ Through extensive simulations it was shown that in addition to TJ formation, structural changes caused by cell differentiation can also be determined from impedance analysis. Specifically, 3D villi formation in a gut-on-chip model resulted in an increase of the cell capacitance of the impedance model. The villi formation was also confirmed by microscopy. Furthermore, the authors discuss the benefits of integrating several small TEER electrodes, instead of the conventional format of using four large electrodes. By using local electrode pairs, nonuniform potential distribution caused by large electrodes can be avoided and a more stable TEER read-out can be obtained.

### 4.2 ECIS

Cell impedance measurements, in many ways similar to the TEER measurements, can also be conducted when cells are grown directly on the surfaces of electrodes. This method offers some benefits, among them localized measurement spots and the possibility to perform cell adhesion measurements. Making use of the localized measurements, Tran et al. designed a chip to investigate how far cell–cell interactions between cancer cells and stromal cells could extend.^128^ In their chip, they used A549 tumor cells and MRC-5 fibroblasts. To investigate the effect of distance, the two cell types were cultured next to each other with a removable fence. On both sides of the separation line, an array of 100 μm diameter Ti/Au electrodes were sputtered onto the glass substrate together with a common large counter electrode on one side. The impedance of the individual electrodes was measured at 10 kHz and normalized with a measurement before cell seeding, to give a so-called *cell index*. Using a cell index is quite common for ECIS measurements in order to assist the data analysis by considering only changes in impedance at a single frequency, which has been thoughtfully selected for the given experiment. This study compared measurements with the fence in place, limiting the cell–cell interactions to indirect contact through soluble factors in the cell media, and after removal of the fence where also direct cell–cell contact was allowed. In combination with adding curcumin, known to significantly inhibit growth of tumor cells, proliferation was measured when the fibroblasts were in close proximity (250 μm apart), while no inhibition was detected when the tumor cells were 3 mm or more away from the fibroblasts.

ECIS using larger electrodes and analysis at fixed frequencies has been shown by several groups. Zhang et al.^55^ incorporated Au electrodes in a stretchable PDMS layer, to measure proliferation of bovine aortic endothelial cells (BAECs) during cyclic stretching, and Wu et al.^124^ measured the anticarcinogen inhibition effect of different drugs on lung cancer spheroids cultured from A549, H1299, or H460 cells. In their work, they revealed a higher resilience to the drugs of the 3D cultured spheroids compared to standard 2D cultures. Pan et al.^133^ also measured drug efficacy on 3D cell clusters in matrigel but using a simplified fabrication scheme with vertically integrated electrodes in a custom-built cell culture chamber. The vertical electrodes were formed by bent solid Au wires simply inserted through the bottom plate of the custom-built setup.

It can be beneficial to run *in situ* microscopy of the cells together with the ECIS measurements, and for this, transparent electrodes, such as ITO thin films, are highly suitable. Both An et al.^17^ and Khalid et al.^127^ have demonstrated that this is a viable option with no indication of cell toxicity.

Similar to the TEER measurements, more information can be gained by collecting data from an impedance spectrum as compared to measurements at a single frequency. Characteristic for ECIS, however, is the ability to deduce the frequency dependent impedance change related to cell adhesion.^141^ Kang et al. made use of this in their flow-speed-dependent cytotoxicity assay chip. They designed a microfluidic system with a stepwise increasing flow speed obtained through a narrowing of the channel width.^64^ Cells were cultured in the whole microchannel and ECIS sensors fabricated from ITO were placed in each flow speed area. This allowed for evaluation of the cytotoxicity of the flushed medium at different flow speeds simultaneously by measuring changes in cell adhesion at different locations along the channel. The functionality was demonstrated with human breast cancer cells (MDA-MB-231) and a 5% ethanol media solution by analyzing the impedance, detachment rate, and death rate for flow speeds of 0–8.3 mm/s. They concluded that the cultured cells were more affected by the ethanol at lower flow speeds, probably due to the longer interaction times between the cells and the toxin.

### 4.3 Field Potential Sensors

MEAs have been established and integrated in multiwell plates for mapping of electrophysiological activity of cells, and the vast majority of works in this field are performed on static cell cultures and thus fall outside the scope of this review article. However, MEAs have also been integrated into perfused cell cultures and organ-on-chip models on a few occasions.

Most commonly, the MEAs are fabricated on solid supports which limits their ability to closely mimic the soft *in vivo* conditions. McCain et al. have demonstrated the use of soft materials as scaffolds to induce structural orientation of engineered tissue^142^ and have shown that it is possible to coat MEAs with gelatin to provide a culture environment supporting human induced pluripotent stem cell-derived cardiomyocytes (hiPSC-CMs).^136^ In their work, they assembled a microfluidic system comprising a polyetheretherketone (PEEK) culture chamber and a commercially available MEA substrate. They measured spontaneous cardiac field potentials under perfused culture conditions and upon pharmacological interventions of isoproterenol, terfenadine, and fexofenadine, which are three drugs known to affect the QT interval *in vivo.* The model reported changes in cardiac beating rate as expected and the authors conclude that the gelatin coating on the MEAs increased cell viability over time without isolating the signal from the extracellular electrodes and, thus, showing the potential of using this sensing method for cells that need a tailored 3D environment.

Along the same lines, to further expand the application area of MEAs Gaio et al. developed MEAs integrated in a flexible PDMS membrane.^135^ The electrode structures comprised TiN electrodes embedded in the PDMS with Al contact pads outside the cell culture area. A total of 12 electrodes were introduced on the membrane having an approximate area of 0.5 mm^2^. Although this does not correspond to a specifically high electrode density, the authors could validate the functionality of the MEAs by recording the electrical activity of hiPSC-CMs plated onto the membrane. They detected a typical signal of 100 μV and argue that these types of MEAs could be used for both stimulation (no available experimental data) and read-out of electrically active cells, such as heart, muscle, and neural cells, in the future. To further explore these possibilities, studies on the effect of wear of the TiN electrodes upon membrane stretching should be addressed.

Neurons or cardiac tissue, which are often studied using MEAs, give rise to relatively high electrical signals that are straightforward to detect. Other groups of cells, such as insulin producing islets, could be more difficult to study because of the significantly lower signal strength of their action potentials. Koutsouras et al. addressed this challenge by preparing Au MEAs that were coated with PEDOT:PSS to reduce the electrode–electrolyte contact impedance and increase the S/N ratio.^18^ Measuring the impedance of the different electrodes (pure Au or coated Au) over the full frequency range, they specifically noted a reduced impedance in the higher frequency range (>100 Hz), which would increase the S/N for action potential detection. On the other hand, they also noted an increased contact impedance for the coated electrodes in the lower frequency range, which would in turn reduce the S/N for the signal from the slow potential, i.e. the signal reflecting cell–cell communication along the tissue construct. Hence, it is important to consider the final application before deciding if this polymer coating is suitable or not. The same group has also explored the use of Pt black electrodes to detect electrophysiological activity of insulin producing islets upon exposure to glucose. Again, they report an improved S/N compared to using standard thin-film metal electrodes.^62^

Zhang et al.^138^ used MEAs to detect spontaneous beating from 3D cardiac cell cultures. To bring the electrodes into contact with as many cells as possible, they integrated two large pillar electrodes at either end of a dog bone shaped culture channel instead of fabricating flat electrodes on the surface. They fabricated the electrodes by adding Pt powder into a PDMS prepolymer matrix and molding the composite into 1 mm tall circular electrodes with a diameter of 300 μm resulting in a total electrode area of ∼0.9 mm^2^ each. The integrated electrodes were used for both stimulation and sensing from the cultured cells. Primary cardiomyocytes obtained from rats were cultured in the system for up to 3 weeks where the pillar electrodes were used to measure the tissue activity and its change upon exposure to isoproterenol, a drug known to affect the beat rate in cardiac tissue.

Through basic analysis on cardiomyocytes, the data can be used as an *in vitro* mimic of the QT interval of the heart’s electrical cycle which is used *in vivo* to evaluate possible pharmacological side effects of new drug candidates.^143^ By applying careful modeling one can obtain detailed information on the activity of the different membrane ion channels involved.^144^

### 4.4 Strain Gauges

The effect of mechanical actuation and external mechanical stimuli on cells and tissue is studied in the field of mechanobiology. Hence, integrated strain gauges have been extensively used to study single cells,^145^ and the interested reader is referred to a recent special issue on the topic.^146^ The integration of strain gauges into OoCs has not yet reached the same level of maturity although it has been presented in the work by Lind et al.^21^ Here, they present a flexible cantilever structure fabricated in PDMS with integrated thin-film electrodes. To be able to stretch the thin-film metal electrodes, they utilized the technique of depositing a microcracked adhesion layer.^147^ Cardiomyocytes were seeded on top of the flexible cantilevers, and the cantilever deflection caused by tissue contraction was studied under exposure to cardiac and cardiotoxic drugs, such as isradipine and the antidepressant desipramine. The noted responses compared well with available *in vivo* data, demonstrating the effectiveness of this approach in generating dose–response curves in a user-friendly setup with reasonable through-put.

### 4.5 Electrochemical Sensors

Another category of analytes in OoC are cell-secreted soluble biomarkers that can be detected using impedance spectroscopy and amperometry methods. There are few examples in the literature of these being implemented in OoCs. Here we have included the relevant ones.

Shin et al. have demonstrated two types of applications of aptamer based sensor platforms: first, to monitor creatin kinease as a marker of damage to cardiac organoids in a heart-on-chip model^80^ and later to monitor changes in cell metabolism induced by the liver-toxic drug APAP^79^ in a liver-on-chip model that included sensors for detecting albumin and glutathione-*S*-transferase-alpha (α-GST). In both works, detection was based on injecting an electrolyte into the chip and measuring the electrochemical impedance. The addition of reagents is not favorable; however, the versatility of aptamers allows other analytes of interest to be detected. Upon antibody–antigen binding, there was an increased resistance of the charge transfer for the [K_3_Fe(CN)_6_]^−3/–4^ redox process, which was introduced via the electrolyte.^79,80^ The same group also measured transferrin and albumin in a liver-on-chip and a bioreactor with integrated disposable magnetic microbeads. They monitored the release of biomarkers under the effect of APAP with an on-chip immunoassay by immobilizing antibodies on the surface of the beads. The magnetic microbeads functionalized with antibodies against transferrin or albumin were captured with a magnet in a reaction chamber, and sample solutions were introduced into the chip. Subsequently, they utilize secondary antibodies linked to the enzyme horseradish peroxidase and the oxidation reaction between H_2_O_2_ and TMB (tetramethylbenzidine). Finally, the oxidized TMB is directed to a detection chamber containing electrodes for amperometric measurements.^81^ Ortega et al. reported a muscle-on-chip system to study the release of interleukin 6 (IL-6) and tumor necrosis factor alpha (TNF-α) under electrochemical and biological stimulation based on screen-printed gold electrodes (SPGEs) with immobilized antibodies. However, analysis with SPGE is performed off-chip, resulting in measurements that are not in real-time. As a final step in their sensing protocol, the SPGEs are removed from the OoC system and placed in a PMMA cell to be processed so that a current related to the amount of IL-6 and TNF-α can be measured by connecting the SPGEs to a potentiostat.^148^

Misun et al. integrated enzyme-based sensors for lactate and glucose in a hanging droplet OoC by immobilizing oxidase enzymes in a hydrogel of cross-linked BSA. Metabolism of human colon carcinoma microtissue was measured in real-time under different culture conditions to record secreted lactate and glucose consumption. Sensors were integrated above the cells on the ceiling substrate in which the hanging droplets that hosted spheroid cultures were suspended. The open design of the hanging droplet chip is beneficial for the integration and modification of the electrodes as the bonding steps that can degrade sensor components are not required. They obtained real-time information on the metabolic state of cancerous microtissue under different cell culture conditions by measuring glucose consumption and lactate secretion.^31^

Amperometric sensors were also used to monitor cell culture conditions in OoCs. Moya et al. integrated amperometric oxygen sensors in a liver-on-chip system using inkjet printed electrodes to allow for local measurements. This approach enabled them to monitor gradual changes in dissolved oxygen concentrations along the channel in a liver-on-chip model using primary cells. Inkjet printing offers the advantage that electrodes can be printed directly onto a substrate and no mask is needed, which reduced the time and cost of sensor production. To demonstrate use of their system, proof of concept measurements were performed with primary human and rat hepatocytes and carbonyl-cyanide-4-(trifluoromethoxy)phenylhydrazone, which increases cell consumption of oxygen.^140^

### 4.6 Luminescent Optical Sensors

Optical sensors have been successfully integrated in microbioreactors and microfluidic systems to monitor pH and oxygen.^86,92,93,97^ However, it is only oxygen sensors that to date have been reported in OoCs. Single point measurements can provide valuable data on the culture conditions inside a microfluidic device as well as information on the oxygen consumption rates (OCRs) of the cells. Several sensor spots are often integrated inside a device so that an estimation of the gradient that forms across the cell culture area can be made. Rennert et al.^33^ developed a liver model using a chip with two parallel chambers separated by a membrane. Two oxygen sensor spots were integrated in each chamber. One of the sensor spots was placed in the inlet and the other near the outlet of the chamber. The sensor used lifetime measurements, based on phase modulation, and the sensor positions allowed a comparison of the conditions in the two compartments as well as an estimation of the oxygen gradient forming along the chambers under perfusion. They measured the OCR of HUVECs and a HepaRG cell layer under static conditions and compared this to measurements under perfusion applied to both or only one compartment. Measurements during vascular perfusion showed the formation of an oxygen gradient resembling *in vivo* conditions.

Another investigation of oxygen gradients inside microfluidic devices using several sensor spots was performed by Zirath et al.^100^ They used four spots to investigate oxygen gradients in 3D hydrogel-based cell cultures, and, for a chip fabricated using an oxygen impermeable material, demonstrated the possibility to control the oxygen levels within the hydrogel by adjusting the flow rate. In a similar experiment, they established a perfusion protocol to measure the OCR of cells in a 2D culture. They compared the OCR of different cell types and seeding densities and could reveal different cell OCRs depending on their adhesion to the substrate. They conclude that oxygen measurements can be used to conduct cell adhesion and biocompatibility studies for different surface treatments.^100^

Another method used to measure the OCR was applied by Prill et al.^34^ They established a culture of hepatic spheroids including oxygen sensor doped PS microparticles in a microfluidic bioreactor. The lifetime based measurement allowed for simultaneous readout of all sensor beads, enabling high throughput measurements. The measured OCR of the cells inside the spheroid was used to evaluate the response of the system to drugs, *e.g*. amiodarone and acetaminophen. This dynamic response revealed important information on the toxin mechanism of action and the presence of transient subthreshold effects of the drugs, which classical end points analyses cannot demonstrate. Later the system was combined with electrochemical glucose and lactate sensors^30^ (cf. Section 4.7).

Optical sensors can be used to assess the 2D distribution of an analyte in a system using a sensor film. Readout with microscopic techniques allows high resolution 2D mapping of the analyte. The 2D oxygen gradient that forms upon cell culture in an oxygen impermeable system can be used to access the OCR of the cells under perfusion. Matsumoto et al.^139^ cultured cells in a microfluidic channel under different flow rates. They monitored the oxygen gradient inside the device with an oxygen sensitive film and simple intensity-based measurements using a microscope. After comparison of the mapped oxygen gradient to a simulation of gradients based on different OCRs, the OCR of the hepatic cell culture was determined. An improvement of intensity-based measurements is possible by using ratiometric methods. Ungerböck et al.^40^ studied the oxygen distribution in microfluidic devices with cell culture by including a sensor matrix with both an oxygen sensitive dye and an inert reference dye at the bottom of the culture area. This enabled ratiometric intensity measurement using a fluorescence microscope and a color camera. They compared the oxygen distribution of cells cultured in monolayers to cells cultured as clusters and found that oxygen gradients arise when cells are cultured in clusters, a feature that could not be detected using single point measurements.

### 4.7 Combination of Different Sensing Principles

In order to fully exploit the potential of in-line analysis in OoC, it is important that several parameters can be measured in parallel. Since not all parameters can be determined with the same sensing principle a combination of different sensing principles is therefore necessary. However, this can be a technological challenge and results in more complex microfluidic devices. Here, we present those OoCs that use more than one sensor type and the achieved benefits.

Combining different types of electrical sensors does not require access to different fabrication techniques, read-out instrumentation, and expertise in different fields of sensing techniques, which is needed when, *e.g*., an electrical sensor is combined with an optical sensor. Hence, combinations of electrical sensors are rather easier to realize.

Maoz et al.^19^ developed a heart-on-chip model with two different types of electrical sensors—TEER and MEAs—that were both designed and fabricated in-house. The system comprised two parallel channels with a porous membrane in-between. On the membrane, human umbilical cord vascular endothelial cells (HUVECs) were cultured to mimic the vessel wall. In the lower channel hiPSC-CMs were cultured, directly on top of the microfabricated MEAs, mimicking the cardiac tissue. By using both sensor types, the authors could follow the disruption of the endothelium upon exposure of the inflammatory stimulus TNF-α and the consecutive increase in beat rate of the myocardium if the drug isoproterenol was introduced into the vascular channel afterward. No change in beat rate could be observed in the cardiac cell culture when isoproterenol was introduced into the channel with an intact endothelium.

Another group drawing upon the advantages of combining more than one electrical sensor type is Qian et al.^130^ In their work, they combined Pt black coated electrodes in a MEA configuration with Au interdigitated electrodes (IDEs) for simultaneous monitoring of electrophysiology and tissue growth of human iPSC-CMs. By combining the two different sensor types, they demonstrate the possibility to decouple the read-out of contraction and electrical activity of cardiac cells. In their work they showed how the platform could be used to electrically stimulate cardiomyocytes and follow cell proliferation and electrical activity for up to 9 days. The decoupling was achieved by exposing the cells to blebbistatin, which is an agent that stops cell contraction without affecting the action potential of the cells. Upon introduction of blebbistatin, the ECIS sensor no longer observed any impedance change induced by the change in cell shape during contraction, whereas the MEA still detected field potential signals. The authors argue that their platform could be important in evaluating negative side effects of new drug candidates, and this work clearly shows the power of combining more than one sensor type in organ models.

A combination of optical and electrical sensors was used by Shah et al.^32^ to control the culture conditions in their device. The authors developed a model of the gastrointestinal human–microbe interface, named HuMiX (human–microbial crosstalk) that consists of three stacked microfluidic chambers. A key feature of this device is the possibility to perfuse each of the chambers individually and establish different oxygen concentrations in the chambers. Aerobic conditions are needed to culture human epithelial cells, whereas anaerobic or aerobic conditions are used in the microbe culture chamber depending on the cultured microbes and experiment. Commercially available oxygen sensor foils were fixated into pockets in the upper and lower chambers using a silicone adhesive and both spots were accessed simultaneously using fiber optics. This allowed for in-line monitoring of the culture conditions in the respective chambers and an estimation of the gradient that formed over the chamber that contained the human epithelial cells. Furthermore, commercially available “chop-stick” electrodes have been used to perform TEER measurements. TEER was used in combination with immunofluorescence microscopy to characterize the cell growth and differentiation inside the device. The TEER measurement was performed as an end-point measurement because the electrodes were not really integrated but rather inserted in ports of the device, which could cause contamination. A real integration of electrodes using microfabrication techniques would eliminate this risk and allow for multiple measurements during the culture. Furthermore, it would allow more elevated electrode configurations, which are advantageous in gaining reliable and comparable measurements as described in section 4.1. Nevertheless, the use of commercially available optical oxygen sensors and TEER electrodes showed that measuring these parameters is essential in controlling the culture conditions of the cells inside a microfluidic device.

**Figure 9:**
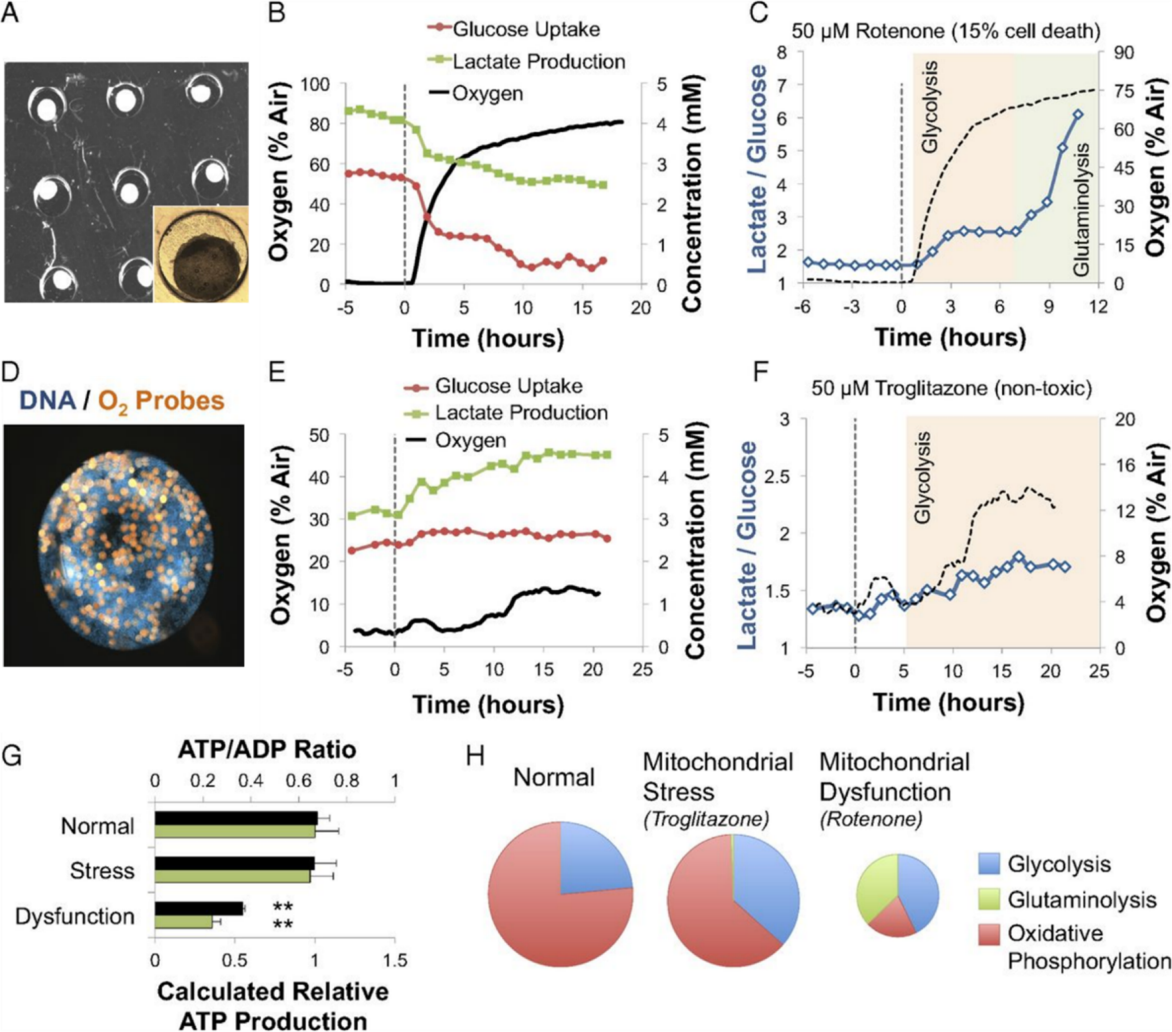
Measurement of oxygen, glucose uptake, and lactate production in a liver-on-chip system (A) treated with rotenone (B) and trogliazone (E). The oxygen consumption of the cells is monitored via luminescent sensor beads embedded directly in the organoid (D). The lactate/glucose ratio together with the measured oxygen concentration indicates the metabolic state of the liver organoids during the treatment (C, F). The ATP/ADP ratio inside the cells under different conditions was predicted based on the measurements and verified with off-line assays (G). The metabolic sources of ATP production are displayed as pie charts with relative diameter for the treated and untreated cells (H) (Adapted with permission from ref (30). Copyright 2016 National Academy of Sciences).

Bavli et al.^30^ showed how a combination of optical and electrochemical sensors can be used to determine the metabolic state of cells during toxicity tests. They combined a liver-on-chip developed by Prill et al.,^34^ which comprised optical oxygen sensors, with electrochemical glucose and lactate sensors. The electrochemical sensors were placed in-line downstream of the cell culture area, whereas the luminescent oxygen sensor beads were incorporated directly in the liver organoids. Monitoring both oxygen and glucose uptake and lactate production simultaneously enabled the metabolic changes of the cultured cells to be observed in greater detail. The authors used the anti-inflammatory drug troglitazone, to induce mitochondrial stress, and the pesticide rotenone, to induce mitochondrial dysfunction, by adding the compounds to the perfused media. Using the real-time data of the integrated sensors they were able to predict the metabolic fluxes and calculate the intracellular adenosine triphosphate (ATP) production (Figure 9). They could confirm their on-chip measurements with established off-line assays. Interestingly, they detected metabolic changes indicative of mitochondrial dysfunction at drug concentrations that were regarded as safe in previous reports.

Another group investigating the use of multiparametric sensing in OoCs is Zhang et al.^43^ In their work, they combined a physical sensing unit and an electrochemical biosensing chip with a liver-on-chip and a heart-on-chip model on a microfluidic circuit board. Electrochemical biosensors were functionalized with antibodies to measure albumin, α-GST, and creatine kinase MB (CK-MB). The physical sensing chip comprised optical sensors to measure pH and oxygen together with an electrical temperature sensor. Oxygen measurement was based on dynamic quenching of an immobilized indicator dye, and pH was detected from absorption measurements of phenol red dissolved in the culture medium. To study systemic effects of acetaminophen, the two organ models were connected via a circuit board and the read-out of the physical and the chemical responses was followed. No changes in the levels of oxygen and pH were measured upon introduction of the drugs (acetaminophen or doxorubicin). The constant oxygen level could mainly be maintained due to the high gas permeability of the PDMS/silicon chip reoxygenating the culture medium from outside. The biosensors showed a dose-dependent increase in α-GST and a dose-dependent decrease in albumin secretion when acetaminophen was introduced, showing the hepatotoxic side effect of this drug. Systems that were treated with doxorubicin showed high values of CK-MB, as expected. In another set of experiments, the metabolic reaction of liver–cancer organoids upon hyperthermia treatment was investigated. This experiment was conducted in a sealed platform with lower oxygen permeability to avoid the reoxygenation from the outside environment. The monitored oxygen and pH values provide evidence on the metabolic state of the organoids and their response to hyperthermia treatments. No significant response of the cell’s metabolism was found below 43 °C.

## 5 Outlook

This perspective review has described various sensor types that can be used to monitor environmental conditions in cell cultures and especially organ-on-chip systems. One of the aims of OoCs is to mimic physiological systems, hence in-line read-out is very important and sometimes even critical to enable rapid adjustments. The last section of this review addresses a few sensor parameters that can provide useful information in OoCs but are not monitored today, for instance reactive oxygen species (ROS), reactive nitrogen species (RNS), CO_2_, and ions such as sodium, potassium, and chloride.

### 5.1 Reactive Oxygen/Nitrogen Species

Monitoring ROS and RNS can provide useful information about cellular responses, for instance, ROS is a byproduct of aerobic metabolism.^25^ In general, measuring ROS and RNS is not straightforward and remains a challenging analytical task. Only species with a relatively long lifetime can be measured, and indeed extracellular selectivity for one species is hard to achieve in itself. Neither ROS nor RNS monitoring has been implemented in OoCs. We have selected reports from microfluidics, because they have the potential to be transferred to OoCs and often similar fabrication methods can be used from a device point of view. We refer to the review by Shi et al.^25^ for an overview on microfluidic devices for ROS detection. Giménez-Gómez et al. fabricated a lab-on-a-chip with an integrated amperometric hydrogen peroxide sensor (H_2_O_2_). H_2_O_2_ was measured with a thin-film gold electrode, although the issue of selectivity is not discussed.^65^ Li et al. utilized downstream amperometric measurements in a microfluidic platform to monitor four long-lived primary ROS and RNS secreted from a cell culture.^149^

ROS and RNS can also be detected using electrochemical biosensors where horseradish peroxidase coated Au electrodes can be used to detect hydrogen peroxide as reported by Matharu et al.^150^ Another example where horseradish peroxidase has been used is provided by Inoue et al. They coated an ITO electrode with osmium-polyvinylpyridine gel polymer containing horseradish peroxidase and placed a PDMS well on top to house cells.^151^ It should be noted that the electrode in the latter example is relatively large, and some miniaturization is needed for the implementation into OoCs.

A different ROS that has been measured with enzymatic biosensor detection is superoxide radical (O_2_^•–^) with either superoxide dismutase (SOD) or cytochrome c (cyt c). SOD has been utilized in combination with a mediator (ferrocene-carboxaldehyde) and applied in a flow cell^152^ or based on direct electron transfer between SOD and an electrode.^153,154^ Additionally, cyt c has been used to make a biosensor for O_2_^•–^,^155^ as well as biosensors for H_2_O_2_.^156,157^ None of the examples mentioned with biosensors utilizing SOD or cyt c are employed in microfluidics, and therefore, miniaturization and optimization are needed before they can be used in OoCs. Furthermore, they have similar issues like other ROS and RNS electrochemical sensors that suffer from poor selectivity in general.

### 5.2 Carbon Dioxide

Oxygen is the main analyte monitored to follow cellular aerobic respiration as it is consumed during the process. Another relevant analyte that is equally accessible is carbon dioxide (CO_2_), which is produced during respiration.^25,158^ The most promising CO_2_ sensors for miniaturization are based on optical pH sensitive layers. The pH-sensitive layer needs to be covered by a gas-permeable membrane or lipophilic layer to avoid interference from other ionic species,^158^ and this challenging integration might be the reason that there are no examples of OoCs with integrated CO_2_ sensors reported yet.

### 5.3 Acidification/pH

Notably, pH is rarely monitored in OoCs although it has been extensively reported in larger microfluidic cell culture systems. In OoCs, the cells are normally continuously perfused with buffer solutions to control the pH of the system. Measuring the pH around the cells could provide useful information about the cell state and cell metabolisms reflected in extracellular acidification, and this calls for sensors with high sensitivity. Conventional glass electrodes are too fragile, challenging to miniaturize, and complicated to build for measuring cell metabolism in microfluidic devices and OoCs.^25^ ISFETs and MOx sensors have great potential for integration in OoC systems to monitor pH as they are commercially available or can be fabricated with standard techniques used in semiconductor manufacturing.^70^ Examples are given below, where MOx sensors and ISFETs have been used to measure cells in microfluidic systems, and we assume similar approaches can be transferred to OoCs. A MOx-based sensor with iridium oxide has for example been used for on-chip measurements of acidification rates of Chinese hamster ovary cells and fibroblast cells,^159^ human brain cancer cells,^67^ and RAW 264.7 macrophages.^66^ Moreover, Mani et al. designed a chip with pH sensitive zinc oxide sensors for investigating circulating tumor cells in blood.^69^ ISFETs have been reported in a microfluidic cell culture system for measuring pH only 10–100 nm away from the cell membrane of tumor cells,^71^ and pH and oxygen consumption in a 2D culture of tumor cells.^160^

Optical sensors are also a promising candidate for measuring the pH in OoC. In contrast to the absorption based sensors discussed above, luminescent sensors can improve the measurement because read-out can be easier realized. Huang et al.^86^ used luminescent pH and oxygen sensors to measure the OCR and the acid extrusion rate of zebrafish embryos in a microfluidic device. They monitored the transition between aerobic and anaerobic metabolism under acute hypoxia. Tahirbegi et al.^93^ integrated luminescent pH and oxygen sensors in a microfluidic device with an algae culture. They showed that they were able to investigate the metabolism of the algae using the sensors and thus the influence of the pesticide diuron on the culture. Lee et al.^97^ presented luminescent sensors in a flow through cell linked to a bioreactor which allowed for continuous online monitoring of pH and oxygen for up to 2 weeks. These examples show the feasibility of pH monitoring in microfluidic systems with cells and the insights on the cell metabolism that can be gained.

### 5.4 Temperature

We foresee a development in the near future whereby electrical sensors are explored for integrated temperature measurements in OoCs. Temperature can be measured via resistive measurements of a conductor, such as Pt, expressing a linear temperature dependence in the relevant range.^161,162^ Conventionally, OoCs are maintained in temperature-controlled incubators during the experiments, but it can still be important to monitor minute temperature changes as they may affect intracellular processes.

### 5.5 Shear Stress

Another application area of integrated temperature sensors involves measuring the flow speed in perfused cultures to monitor shear stress levels. The flow, and hence shear stress, can be measured by integrating a standard suspended thermal conductivity detector in the microfluidic channel. In the detector, a resistive path is heated through joule heating. The convective heat loss when exposed to a liquid flow is proportional to the flow velocity, which can be detected as a change in resistance of the temperature-dependent resistive path. This has been demonstrated by Booth et al., in which they cultured b.End3 cells in a microfluidic chip with four channels of different dimensions.^119^ By integrating thin-film electrodes to monitor both the shear stress levels and TEER of the cultured barrier, they could observe that barrier tightness increased with increasing shear stress levels, as expected.

### 5.6 General Outlook

Finally, increased attention to the development of conducting polymers to enable 3D printed sensors, improved cell/electrode contact and fabrication of flexible substrates with integrated electrical sensors is a foreseen trend in the field. Some reports have been discussed above, using *e.g*. carbon black and Pt black^20,130^ and conducting polymer scaffolds capable of both supporting cell proliferation, enhancing tissue function, and serving as the read-out tool for monitoring cell growth.^163^ Flexible electronics may also enable field potential and cell impedance sensing of nonplanar cell models. Although not in fully integrated OoCs, 3D electrophysiological read-out of single organoids and spheroids using flexible MEAs wrapping around and conforming to the surface of the cell culture has been demonstrated.^164,165^ With the growth in bioelectronics research, we expect conducting polymers and flexible electronics to become one of the main research areas within the OoC field in the coming years.

## 6 Conclusion

This review article discusses integration of three different sensors types; electrical, electrochemical, and optical sensors into OoCs, and we have tried to give a comprehensive understanding of the basic sensing principles and how the sensors can be integrated. From the review, it is clear to see that each of the discussed sensors has its own benefits and limitations so it is important to carefully consider these before choosing which sensor to integrate in the development of a new OoC. It should also be remembered that often different sensor types can be used to monitor the same analyte. Herein, choosing the most beneficial sensor depends not only on the best sensor performance but also on the integration and compatibility with other sensors applied.

It can be noted that electrical sensors are to date more commonly integrated in OoCs compared to the other two sensor types discussed in this review, i.e. electrochemical and optical sensors. This is most probably due to their more straightforward integration of miniaturized electrodes in microfluidic systems. Electrochemical biosensors rely on the same basic structure as electrical sensors, i.e. integrated electrodes enabled either by insertion of metal wires or thin-film deposition, but electrochemical biosensors also require the addition of a biological recognition element. Often, the sensors are designed around enzymatic processes that catalyze an analyte into an electrochemically active product, and often these enzymes are affected by external factors, such as temperature and pH, making their application more complicated in OoCs. Optical sensors are less common because they require an optical read-out system which can be more complicated than the electronics for the electrical sensors. In addition, the sensor chemistry requires knowledge in synthetic chemistry to tailor indicator dyes and polymer materials. On the other hand, luminescent sensors enable contactless measurement and do not need a reference element. A major advantage of optical oxygen sensors is that the measurement does not consume the analyte, unlike amperometric oxygen sensors. This is particularly important in the μL volumes applied in OoCs.

A trend observed during the last couple of years is the development of OoCs integrating more than one sensor type, and this is a development we foresee to continue. Integrating multiple sensors allows for increased information output and an increased robustness of the model as internal cross-checks and calibrations can be included. It does, however, come with the costs of increased complexity of the OoC and could even possibly lead to cross-talk between the sensors.

In summary, we have found that there is still potential for further development of integrated sensors in OoCs, and we expect to see this development as integrated sensors become more reliable and available and when their many advantages compared to off-chip assays are fully appreciated. In fact, exploring new sensors is a research field of its own. Sensor research and development requires expertise in electrical engineering, material science, microfabrication, synthetic chemistry, and biochemistry. Hopefully, this review will help realize further advances in the integration of sensors in OoCs.
